# Evolutionary Constraint and Disease Associations of Post-Translational Modification Sites in Human Genomes

**DOI:** 10.1371/journal.pgen.1004919

**Published:** 2015-01-22

**Authors:** Jüri Reimand, Omar Wagih, Gary D. Bader

**Affiliations:** The Donnelly Centre, University of Toronto, Canada; Yale School of Medicine, UNITED STATES

## Abstract

Interpreting the impact of human genome variation on phenotype is challenging. The functional effect of protein-coding variants is often predicted using sequence conservation and population frequency data, however other factors are likely relevant. We hypothesized that variants in protein post-translational modification (PTM) sites contribute to phenotype variation and disease. We analyzed fraction of rare variants and non-synonymous to synonymous variant ratio (Ka/Ks) in 7,500 human genomes and found a significant negative selection signal in PTM regions independent of six factors, including conservation, codon usage, and GC-content, that is widely distributed across tissue-specific genes and function classes. PTM regions are also enriched in known disease mutations, suggesting that PTM variation is more likely deleterious. PTM constraint also affects flanking sequence around modified residues and increases around clustered sites, indicating presence of functionally important short linear motifs. Using target site motifs of 124 kinases, we predict that at least ∼180,000 motif-breaker amino acid residues that disrupt PTM sites when substituted, and highlight kinase motifs that show specific negative selection and enrichment of disease mutations. We provide this dataset with corresponding hypothesized mechanisms as a community resource. As an example of our integrative approach, we propose that *PTPN11* variants in Noonan syndrome aberrantly activate the protein by disrupting an uncharacterized cluster of phosphorylation sites. Further, as PTMs are molecular switches that are modulated by drugs, we study mutated binding sites of PTM enzymes in disease genes and define a drug-disease network containing 413 novel predicted disease-gene links.

## Author Summary

Individual human genomes differ in numerous and infrequent small-scale changes such as single nucleotide variants. Understanding the biological role of variation and impact on phenotypes such as physical appearance or disease risk is an important challenge. We studied human variation of post-translational modification (PTM) sites spanning >11% of protein sequence. PTMs are chemical modifications of protein residues that extend protein functions and regulate many cellular processes. We found that PTM sites are specifically conserved among humans, indicating that these sequence regions are particularly important for human physiology. We confirm this observation by carefully studying other factors of genome variability, concluding that human PTM sites are broadly constrained in biological contexts. PTM sites are also significantly enriched in disease mutations, thus we can better understand disease genetics by analysing PTMs. We highlight 152 genes where disease mutations significantly accumulate in PTM regions, and integrate these with pharmacological information of PTM enzymes to predict new drug candidates to diseases. As an example, we propose a novel mechanism to *PTPN11* mutations implicated in Noonan syndrome. This work aids understanding of the selective forces acting on protein-coding genome sequence and provides an integrative framework for predicting variant function in population and disease.

## Introduction

Decreasing sequencing costs have led to unprecedented opportunities to explore human genomes [1, 2]. Linking genome information to molecular mechanism and resulting phenotype, including disease, is a key aim of human genetics that is hindered by complex patterns of inter-individual variation [3]. Protein-coding variants found in genome-wide sequencing and association studies are often scored for functional impact using population frequency, evolutionary sequence conservation and physicochemical amino acid properties [4]. However other intrinsic protein features are functionally important. For example, physical interfaces of protein-protein interactions harbor disease mutations [5].

Post-translational modifications (PTMs) are biochemical alterations of amino acids that extend the functional repertoire of proteins. PTMs regulate structural confirmations of proteins, protein-protein interactions and cellular signal transduction central in development and cancer. PTMs are specific to types of amino acids. For instance, phosphorylation affects serines (S), threonines (T), and tyrosines (Y), acetylation and ubiquitination occur on lysines (K) and methylation occurs on lysines (K) and arginines (R). Often PTMs involve reversible reactions mediated by systems of reader-writer-eraser enzymes that recognize short linear motifs in substrate proteins [6, 7]. We hypothesize that genetic variants in PTM regions add and remove molecular interaction sites and cause rewiring of protein networks that impact phenotype with potentially deleterious outcome.

To investigate this hypothesis, we integrated human genome variation data and experimentally confirmed human protein PTMs. We show that PTM-associated protein-coding regions are significantly less variable among humans, independent of major known sources of variation, and also are more likely to harbor disease mutations. Genomic, pathway and network analyses support this observation across a diverse collection of sites, proteins, and processes, demonstrating the value of PTM site integration in discovery of functional genome variation.

## Results

### Rare variation and variant depletion indicate negative selection in PTM regions

**Figure 1:**
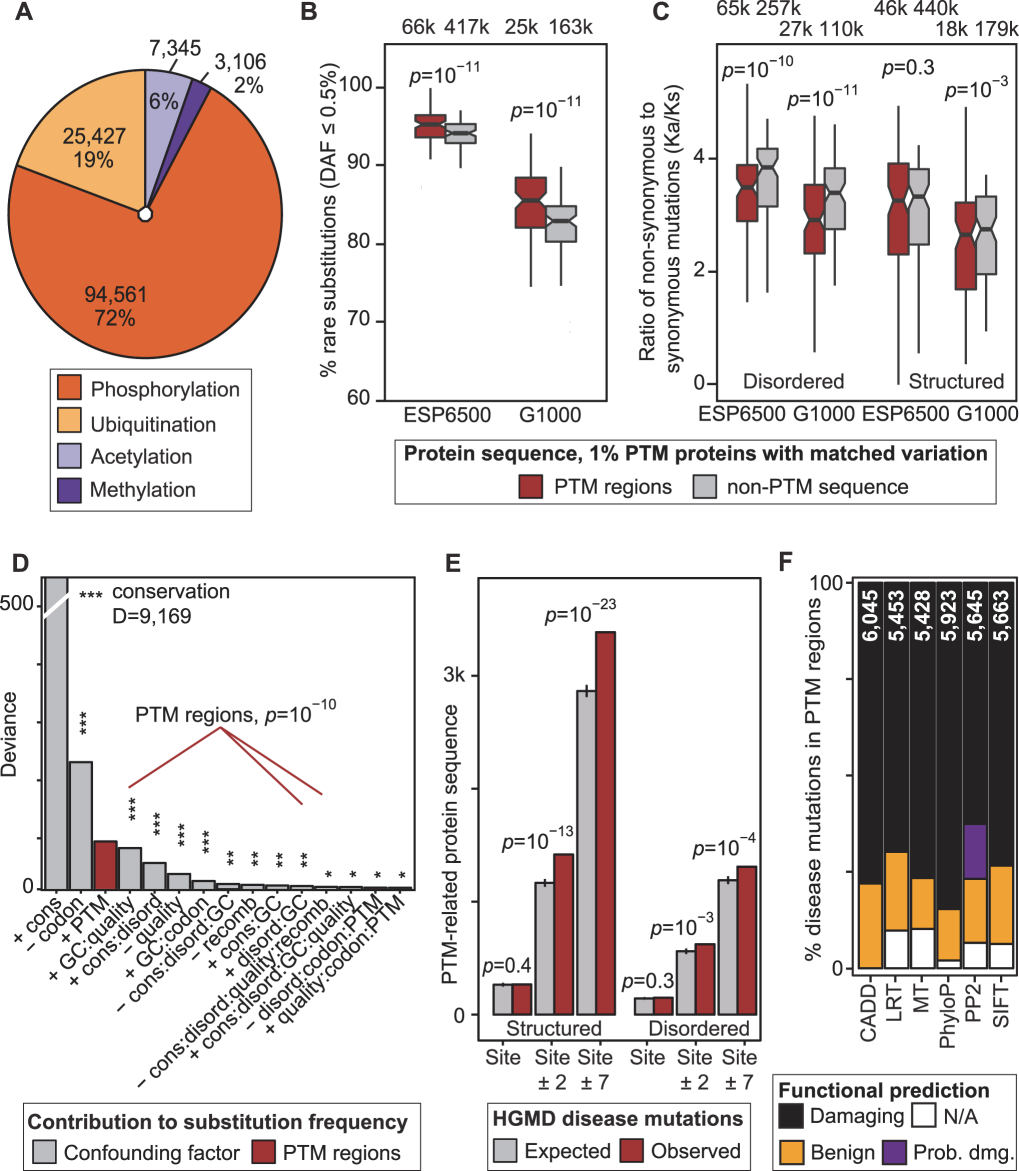
Negative selection of post-translational modification (PTM) regions in human genomes and importance in disease. **(A)** ∼130,000 experimental PTM sites of four types were merged into ∼55,000 PTM regions. **(B-C)** Specific negative selection in PTM regions is apparent in relatively higher frequency of rare substitutions and lower ratio of non-synonymous variants to synonymous variants (K_a_/K_s_). Boxplots represent comparisons of PTM and non-PTM sequence in 100 bins of proteins with matched tolerance to variation. **(D)** Negative selection of PTM regions is a distinct evolutionary trend not confounded by other genomic factors. PTM-associated predictors are shown in red with the variable corresponding to PTM regions ranked third after conservation and codon bias. **(E)** Known disease mutations from the HGMD database are enriched in PTM regions. While central PTM sites appear at an expected mutation rate in this global analysis, amino acid weighted sampling reveals an enrichment of PTM sites (S15 Fig.). **(F)** Disease-associated substitutions in PTM regions are often predicted to be benign by mutation function predictors. Total number of variants scored by each method is shown on each bar.

We first investigated global trends of PTM-associated variation and focused on four modification types with the most experimental data for human proteins. Phosphorylation is the best-described PTM with important roles in core cellular processes such as cell cycle, as well as developmental and cancer pathways [8]. Methylation and acetylation are modifications primarily involved in epigenetics and regulation of chromatin state [9], and ubiquitination is most commonly known as the signal for protein degradation [10]. We collected 130,439 experimentally verified PTM sites from public databases [11–13] with phosphorylation representing 72% of all sites (Fig. 1A). We added ±7 flanking residues around PTM sites to account for short linear motifs and merged overlapping sequence into 55,543 PTM regions. PTM regions are abundant in the proteome, representing 11% of total protein sequence and involving ∼66% of proteins, with more than 25% of proteins having five or more PTM regions (S1–S2 Fig.).

To evaluate the importance of PTM regions, we sought region-specific signs of selection in two population genomics projects, the Exome Sequencing Project [1] (ESP6500) and the 1000 Genomes Project [2] (G1000). We studied non-synonymous single nucleotide variants (SNVs) resulting in amino acid substitutions. Selection was inferred with two complementary criteria, proportion of rare substitutions (Derived Allele Frequency, DAF≤0.5%), and ratio of non-synonymous to synonymous variants (K_a_/K_s_). We carried out paired comparisons of PTM regions and non-PTM sequence in 100 bins of proteins with matched substitution rates. We found that proteins with PTMs are significantly less variable in general (S3 Fig.), and thus we restricted our analysis to 12,495 proteins with PTMs to avoid systematic biases. Variation in PTM regions and non-PTM sequence comprised 77,819 and 493,619 unique substitutions, respectively. We found that PTM regions have significantly more rare substitutions compared to non-modified protein sequence (*p*<10^−10^, paired Wilcoxon test, Fig. 1B). PTM regions also have lower K_a_/K_s_ ratio compared to matched non-PTM sequence (Fig. 1C). Significantly lower K_a_/K_s_ ratio is apparent in disordered protein sequence (*p*<10^−10^), while structured protein sequence shows a mildly significant difference (*p* = 3.9×10^−3^ for G1000, *p* = 0.32 for ESP6500).

To validate the robustness of our observations, we repeated the analyses with adjusted parameters, and subsets of sites and proteins. First, PTM regions are also constrained when all 18,671 proteins are considered (S4 Fig.), and when PTM sites are restricted to high-confidence findings with multiple independent publications (S5 Fig.). Negative selection of PTM regions is observed across bins of proteins with similar substitution frequencies, indicating that PTM constraint is independent of local variation rate (S6–S7 Fig.). Disordered PTM regions are particularly significant, highlighting areas of constraint in less conserved sequence regions. Rare PTM enrichment is apparent across the gene expression intensity spectrum of the Human Tissue Expression Atlas [14] (S8 Fig.), suggesting that our results are not influenced by increased sensitivity of PTM mapping experiments to abundant proteins. PTM regions also show increased proportion of rare variants when different DAF thresholds are considered, and single alleles show the strongest enrichment (*p* = 6.3×10^−16^, S9 Fig.). Rare variant enrichment and lower K_a_/K_s_ ratio are confirmed in African and European cohorts of the ESP6500 dataset (S10 Fig.). PTM-specific enrichment of rare substitutions is also significant in protein residues recently diverged between human and chimp (S11 Fig.). Thus, analysis of ∼7,500 protein-coding genomes shows that PTM regions are less variable than variation-matched protein sequence and undergo specific negative selection.

### PTM constraint is distinct from other sources of genome variation

We next sought to verify that PTM-specific negative selection is independent of potentially confounding factors. We used logistic regression models to estimate regional probabilities of rare substitutions given PTM regions as well as six additional sources of variation as predictors, including conservation scores from protein alignment of 100 vertebrates [15], GC nucleotide content, sequencing depth at detected substitutions, predicted protein disorder by DISOPRED2 software [16], recombination rate from the IMPUTE2 software [17], codon usage, and all statistical interactions of these variables. Deviance analysis confirms that PTM regions are significant positive predictors of rare substitutions that cannot be replicated by any combination of other factors (Fig. 1D). In the ESP6500 dataset, PTM regions represent the third strongest predictor of rare substitutions after conservation and codon bias (*p*<6.1×10^−11^; Chi-square test), and in the G1000 dataset as the fourth strongest after GC content (*p*<2.3×10^−15^; S12 Fig.). Other factors support our models: for instance, while higher conservation positively associates with rare substitutions, PTM regions have relatively more rare variants than matched non-PTM protein sequence across the conservation spectrum (S13 Fig.). Thus our analysis highlights specific evolutionary forces on PTM regions that cannot be estimated from conservation and major known factors relevant to genome variation.

### PTM regions harbor abundant disease variants and challenge variant impact prediction

The relative depletion of inter-individual variation in PTM regions suggests that corresponding substitutions are often deleterious. In agreement with this, analysis of ∼51,000 disease-associated non-synonymous SNVs collected in the Human Gene Mutation Database [18] (HGMD) shows their over-representation in PTM regions. PTM regions are affected in 913 disease genes with substitutions in 4,696 protein residues (4,055±88 expected), comprising a significant mutation enrichment in structured as well as disordered protein regions (*p* = 7.1×10^−24^ and *p* = 7.3×10^−5^ respectively, Fisher’s exact test, Fig. 1E). PTM-associated disease mutations are also over-represented when substitutions with multiple disease annotations are considered (S14 Fig.). This confirms earlier analyses of PTM mutations in inherited disease and cancer by our group and others [19–21]. Our dataset includes 503 substitutions that replace 418 central modified residues, leading to hypotheses of disrupted PTM signaling in disease. The number of direct PTM substitutions affecting modified residues is statistically expected given all protein sequence (Fig. 1E), potentially due to small number of such substitutions. However, substitutions in central modified residues are more frequent relative to residues of matched amino acids (S15 Fig.), indicating their importance in disease. For example, phosphorylated residues are more often substituted than serines, threonines, and tyrosines in general (*p*≤1.3×10^−7^). The remaining PTM-associated (flanking region) mutations may function via other mechanisms such as interference with functional short linear motifs involved in signal transduction, studied below.

To further evaluate the functional impact of PTM-associated disease mutations, we characterized corresponding protein substitutions using six state-of-the-art computational methods [22–27]. Between 15–30% of known disease mutations in PTM sites are not scored, or are predicted benign by tools such as PolyPhen2, SIFT, and CADD (Fig. 1F). As sequence conservation is an important variable in these methods, such predictions tend to underestimate the functional importance of disordered protein sequence that is less conserved (S16 Fig.) and enriched in PTMs [28–30] (*p*<10^−300^, OR = 2.24, S17 Fig.). Comparison of functional predictions from PolyPhen2, SIFT and CADD software shows that population variants and disease mutations in disordered regions are systematically less likely to be predicted deleterious than in non-disordered regions (S18 Fig.). Predicting impact of coding variants will therefore benefit from integration of PTM region information.

### PTM constraint is observed across tissue-specific proteins and cellular processes

To understand the extent of evolutionary constraint of PTM regions, we next analyzed groups of proteins with diverse functional annotations. In each group we compared PTM regions with non-PTM protein sequence of that group, thus considering regional and process-specific differences in variation. To reliably estimate expected substitutions in structured and disordered sequences, we used logistic regression models with protein disorder as a confounding factor.

**Figure 2:**
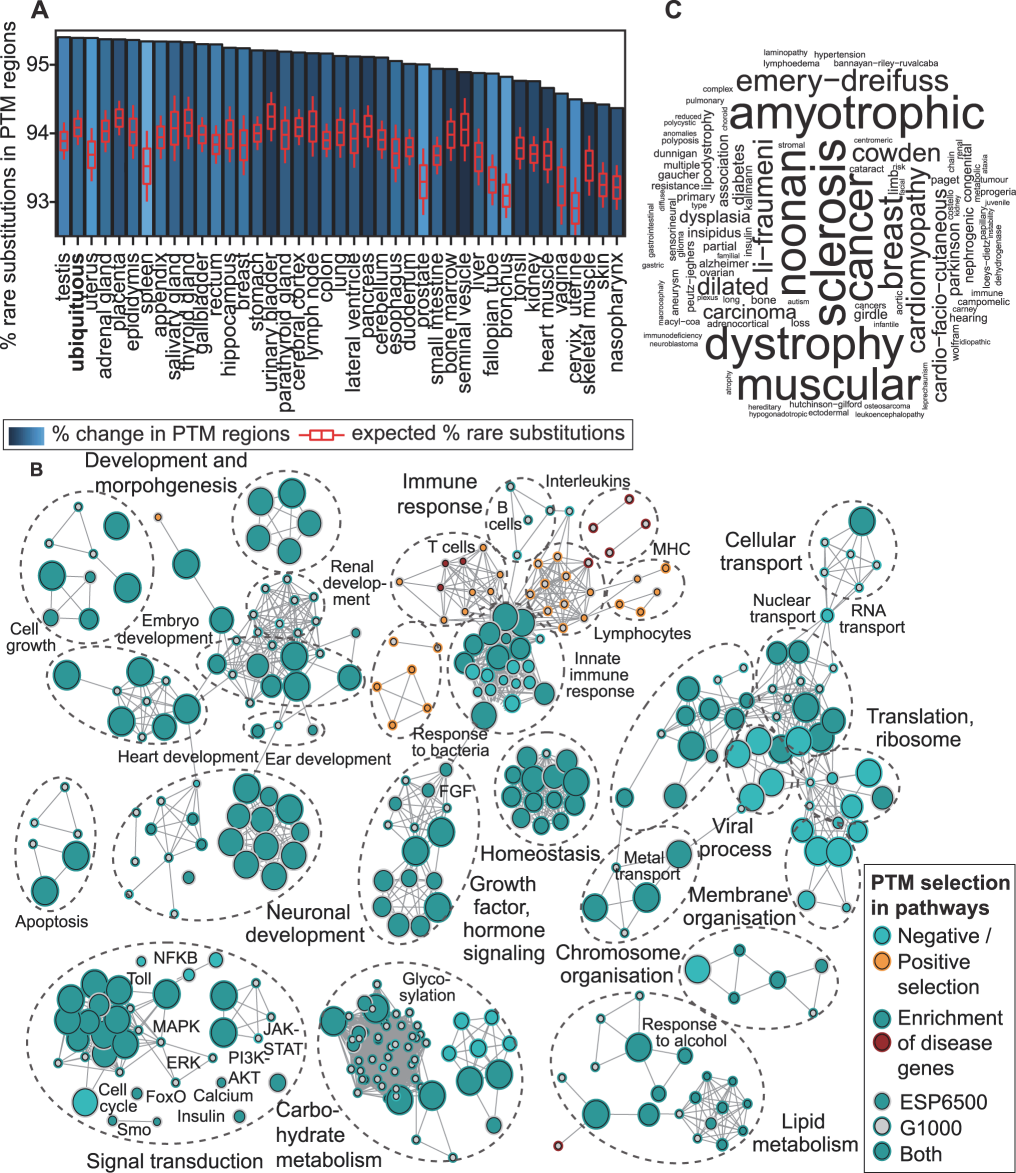
Biological context of evolutionary constraint in PTM regions. **(A)** Negative selection of PTM regions is apparent across human tissues and ubiquitously expressed genes, as 90% of tissue-specific groups of proteins have significantly more rare substitutions in PTM regions. Tissues are ranked by proportion of rare substitutions in PTM sites, and expected proportions in the entire protein group are shown in red boxplots. **(B)** Pathway analysis visualised as an enrichment map reveals 400 biological processes and pathways with significant PTM-specific selection (FDR *p*<0.05). Most processes (90%) show negative selection in PTM regions and ∼75% of processes are also over-represented in PTM-associated disease genes. Nodes indicate processes and pathways and edges show overlaps in annotated genes. Selection in the two population datasets is indicated by node and edge colors (light blue and orange for pathways with negative and positive PTM selection, respectively; dark blue and red for PTM-selected pathways with disease association). **(C)** An example of PTM-associated disease substitutions enriched in significantly selected pathways. The Gene Ontology process of protein modifications (GO:0031401) is enriched in PTM-specific mutations of a wide range of diseases. Word size corresponds to disease annotation frequency.

First we evaluated PTM constraint across human tissues by comparing proportions of rare substitutions in PTM and non-PTM regions in the ESP6500 dataset. We retrieved 44 tissue-specific signatures of protein expression from the Human Protein Atlas [31] and defined a category of ubiquitous proteins (expressed in ≥18 tissues). We found that ubiquitous proteins and 90% of tissue-specific protein groups are enriched in rare substitutions in PTM regions (FDR *p*<0.05, likelihood ratio test, Fig. 2A, S19 Fig.). Top-ranking tissues comprise human reproductive organs such as testis and placenta where gene expression is rapidly evolving [32]. Specific selection against PTM substitutions in the background of rapid evolution suggests that PTM regions control early and central aspects of tissue development and homeostasis [33].

To investigate the physiological function of PTM constraint, we studied groups of proteins annotated to 9,084 biological processes and pathways. Analysis of rare substitutions revealed 400 processes and pathways with significant variation bias in PTM regions, whereas 90% of these processes (359) are enriched in rare variants (FDR *p*<0.05, Fig. 2B, S20–S21 Fig.). The major functional themes with PTM-specific constraint include both rapidly evolving and conserved processes: immune response, embryonic development, brain and nervous system development, heart and renal function, lipid and carbohydrate metabolism, as well as multifunctional signal transduction pathways (*e.g.* PI3K-AKT, MAPK, FGF). While the immune system is generally constrained in PTM regions, we find a few immune-related processes with positive selection of PTM regions, including proteins related to bacterial response, T-cells and the major histocompatibility complex. Interestingly, ∼75% of processes with significant PTM-specific selection are also enriched in disease genes with PTM mutations (FDR *p*<0.01, n = 287; Fisher’s exact test). For instance, proteins annotated to the Gene Ontology term for post-translational modifications are enriched in PTM-associated disease mutations, including cardiomyopathies, diabetes, sclerosis, and cancer (Fig. 2C). As the ESP6500 dataset comprises patient cohorts of heart, lung and blood disorders, phenotypic analysis of rare PTM substitutions in these pathways may reveal novel disease genes and risk modifier variants. Together, these analyses show that PTM constraint and associated disease mutations are widely distributed in genomic and functional context.

### PTM constraint is strongest in central modified residues and PTM clusters

Next we studied substitutions in PTM regions relative to their potential biochemical outcome by measuring selection of distinct types of PTMs and different regions around PTM sites. To account for potential biases arising from variable codon redundancy of specific amino acids modified in different PTMs, we implemented a permutation strategy that proportionately samples relevant amino acids from background protein sequences.

**Figure 3:**
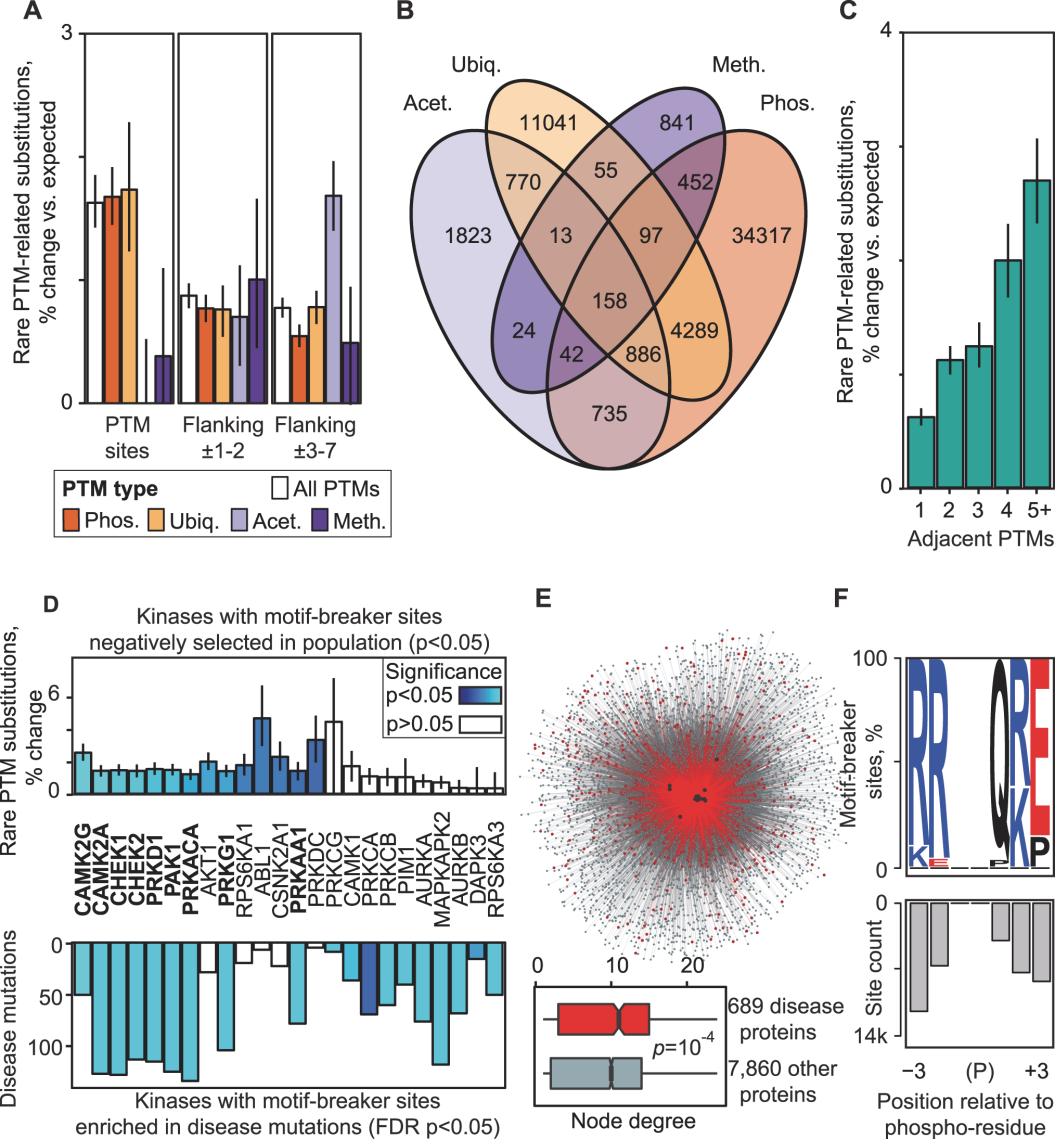
Biochemical consequences PTM variation. **(A)** Negative selection of PTM regions is apparent in different modification types, in central residues modified by PTMs (left) and in flanking regions. **(B)** PTM regions often contain multiple types of modifications. **(C)** Negative selection is stronger in regions with clustered PTMs. **(D)** Variation analysis of kinase binding motifs reveals 24 kinases whose motif-breaker sites are negatively selected in the population (14 kinases), enriched in PTM-specific disease mutations (19 kinases) or both (9 kinases, shown in boldface). Motif-breaker sites are protein residues that disrupt kinase binding motifs when substituted. **(E)** Network of kinase-substrate interactions mediated by motif-breaker sites of the 24 kinases. Disease gene interactions are shown in red and black dots represent kinases with significant motif-breaker sites. Boxplot shows that disease genes have more interactions with motif-breaker sites than other proteins. **(F)** Protein residues highlighted as motif-breaker sites of the 24 kinases, shown relative to PTM site. Motif-breaker sites accumulate within 3 residues and are enriched in R,K,Q,E amino acids. Expected values from amino acid weighted permutations are shown with error bars indicating ±1 s.d.

Analysis of rare substitutions in PTM regions indicates that central post-translationally modified residues are under the strongest negative selection (Fig. 3A, S22 Fig.). This is expected as such substitutions clearly disrupt PTMs and potentially impact PTM-dependent pathways. Selection is also apparent in the flanking sequence even when central amino acids are not considered, suggesting involvement of the electro-chemical environment and short linear motifs of reader-writer-eraser enzymes. This holds true for phosphorylation as well as other PTM types. Although the statistical significance of selection in acetylation and methylation sites is weaker due to fewer sites and substitutions, constraint in their flanking sequence indicates the presence of functionally important residues. As less is known about the sequence specificity of non-phosphorylation PTMs, deeper analysis of constrained PTM regions is needed.

More than 50% of PTM regions comprise multiple modified residues and regions with different PTM types are not uncommon (Fig. 3B, S23 Fig.). We binned residues in PTM regions according to the number of consecutive PTM sites in adjacent sequence. We found that PTM regions with a higher concentration of modified residues are under stronger negative selection according to enrichment of rare substitutions (Fig. 3C, S24 Fig.). Such PTM clusters may reflect complex signaling cascades, for example multi-phosphorylation switches involved in cell cycle control [34], or histone tail modifications where a combinatorial PTM code determines open and closed chromatin states [9]. Substitutions in PTM clusters are more likely to disrupt existing PTM sites or create new sites by adding or removing modifiable residues or critical components of motifs. Together, these data highlight the importance of flanking sequence and suggest the presence of functional elements that regulate PTM interactions.

### Sequence motif analysis in PTM regions reveals 187,000 motif-breaker sites that disrupt modification sites and relate to disease

To further investigate the variation in PTM regions, we focused on kinase signaling, as the human kinome has the most reliable information on substrate specificity [35, 36]. Kinases are known to recognize short linear motifs in flanking sequence of approximately ±7 residues around phosphorylated residues [37]. We scanned 95,021 experimentally confirmed phosphosites for 124 human kinase ligand motifs and predicted a high-confidence set of kinase target sites in flanking sequence using our MIMP software [28][Wagih, Reimand, Bader, *submitted*]. Simulated mutations of these sites identified 61,178 amino acid residues in 81% of phosphoproteins that would dramatically disrupt motifs and lead to loss of signaling when substituted (≥4-fold decrease in binding score). These high-confidence motif-breaker sites cover 7% of PTM regions and contain 366 substitutions annotated to diverse human diseases (S25 Fig.). When also considering direct substitutions of modification sites for phosphorylation and all other available PTM types, we predict 186,704 residues important for PTMs, including mechanistic hypotheses for 863 (14%) of PTM-related disease mutations.

Next we performed kinase-specific analyses of motif-breaker sites by proportionally sampling equal numbers of matched residues from all protein sequence. We found that motif-breaker sites in motifs of 14 kinases are significantly constrained in the population (*p*<0.05, permutation test; Fig. 3D, top panel). Similar analysis revealed 19 kinases whose motif-breaker sites are enriched in disease mutations (FDR *p*<0.05, Fisher’s exact test; Fig. 3D, bottom panel, S26 Fig.). Nine kinases are shared between the two groups, highlighting their importance in signal transduction networks. Top-ranking kinases such as AKT1, CHEK2, and ABL1 regulate core cellular processes of growth and proliferation, and are well-studied cancer drivers according to the CancerGenes database [38]. Members of the calcium-dependent CAMK kinase family involved in neuronal function are also apparent. The site-specific phosphorylation network of the 24 significant kinases includes 7,858 proteins, 69,248 kinase-target interactions and 35,253 motif-breaker sites, whereas PTM-associated disease genes are more central to this network (*p* = 1.6×10^−4^, Wilcoxon test; Fig. 3E). Most of significant motif-breaker sites of the highlighted kinases occur within ±3 residues of the modified residue and involve arginines (R), glutamines (Q), lysines (K), and glutamates (E) (Fig. 3F, S27 Fig.). As we only cover ∼25% of the human kinome with high-confidence motifs, characterization of further kinase binding specificities is likely to reveal additional motif-breaker sites.

In summary, kinase motif analysis reveals negatively selected motif-breaker sites in PTM regions that likely participate in essential cellular signaling and interaction networks. In contrast, frequent disease mutations substitute motif-breaker sites and potentially abolish kinase binding, causing network rewiring. Our dataset of predicted motif-breaker sites is a useful resource for integration into variant interpretation software.

### Analysis of disease mutations in PTM regions reveals a candidate mechanism for Noonan Syndrome and a network of drug-gene-disease interactions

**Figure 4:**
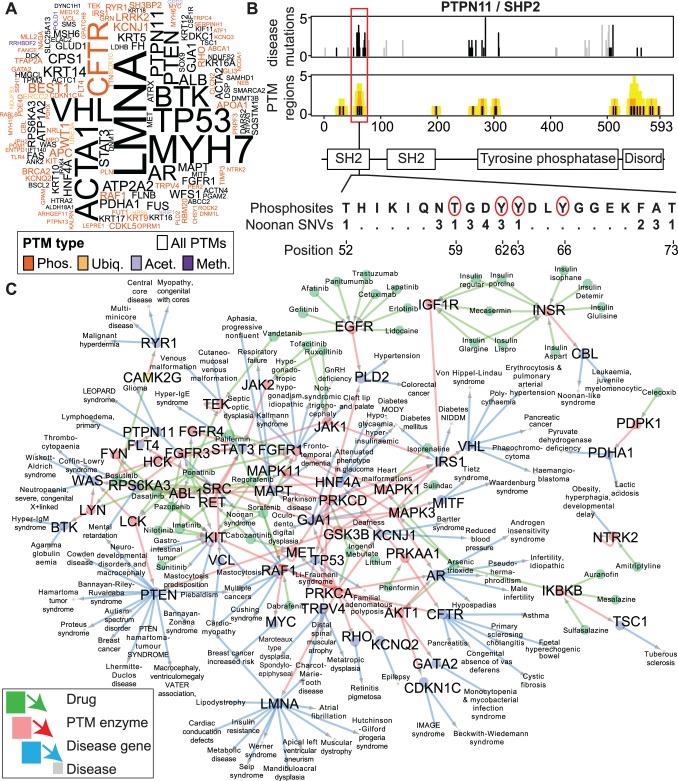
Enriched disease mutations and drug interactions of PTM regions. **(A)** Wordcloud summarizing 152 disease genes with significant enrichment of PTM SNVs from ActiveDriver analysis (PAD list, FDR *p*<0.05). Letter size shows number of PTM mutations in disease. **(B)** An example of a disease gene from ActiveDriver analysis. The *PTPN11* gene encoding the protein phosphatase SHP2 includes a Noonan syndrome-associated mutation hotspot in the SH2 domain of the protein. ActiveDriver shows that the 23 mutations significantly coincide with a cluster of poorly characterised phosphorylation sites (red circles), predicting a disease mechanism of aberrant protein activation. **(C)** Drug-protein-disease network shows PAD genes with PTM mutations whose upstream enzymes are known and druggable with approved pharmaceuticals, highlighting candidates for drug repurposing screens. Only experimentally predicted enzymes bound to significantly disease-mutated PTM sites are shown.

To identify hotspots of disease mutation in PTM regions, we used our ActiveDriver mutational significance model [19] that evaluates enrichment of mutations in protein active sites. This analysis assumes that unexpected co-occurrence of mutations in PTM regions suggests a mechanism involved in disease. We found 152 high-confidence genes with evidence of PTM-associated disease (PAD) where 2,282 disease-annotated substitutions in corresponding proteins are significantly enriched in PTM regions (FDR *p*<0.05 from ActiveDriver, Fig. 4A). Although phosphorylation is the most abundant PTM in our dataset, mutations in 47% of PAD genes affect multiple PTM types, suggesting complex modification mechanisms. The PAD gene list relates to a diverse set of human diseases, including cardiovascular (*LMNA, MYH7*), cystic fibrosis (*CFTR*), diabetes (*HNF4A, IRS1*) and migraine (*ATP1A2*) (S28 Fig.). Cancer genes are also over-represented in agreement with our pan-cancer mutation analyses [19, 28] (31 genes, p = 1×10^−19^). Several genes are known to have PTM-associated disease mechanisms and thus support our analysis. For example, hyper-phosphorylation of Tau proteins is implicated in Alzheimer’s disease [39], and ActiveDriver predicts the corresponding *MAPT* gene as significantly enriched in PTM-related substitutions (FDR *p* = 0.0011). Our predicted list of PAD genes serves as a good starting point for investigating PTM mechanisms in disease.

To exemplify the PAD gene list, we studied the tyrosine phosphatase PTPN11 where substitutions lead to the congenital Noonan Syndrome, a developmental disorder [40]. Half of these substitutions affect an SH2 domain and aberrantly activate the protein by disrupting its auto-inhibitory interaction [41]. We predict that these disease mutations significantly coincide with a phosphorylation cluster (FDR *p* = 0.010 from ActiveDriver, 23 substitutions; Fig. 4B). While no detailed studies exist, ∼30 proteomics screens indicate PTMs in the region according to the PhosphositePlus database [13] (e.g. ref. [42]). Although SH2 is known as a reader domain that interacts with phosphorylated sites [6], phosphorylation of the domain may inhibit its interactions [43]. Thus, we propose that substitutions in Noonan syndrome aberrantly activate the PTPN11 phosphatase by disrupting a phosphorylation-mediated auto-inhibitory loop of the SH2 domain. This example illustrates the integration of PTM information and genetic mutations to predict novel experimentally testable hypotheses of disease mechanisms.

PTM enzymes are well-established drug targets [44, 45]. To investigate novel interactions between PTM-associated drugs and diverse human diseases, we studied PTM mutations in the significant gene list predicted by ActiveDriver. We aimed to discover secondary drug-gene interactions that are not apparent when analyzing drug interactions with known disease genes, but become apparent when studying the post-translational modification networks of disease genes. In particular, many disease mutations in this list affect PTM sites with experimentally verified upstream PTM enzymes, suggesting that disease mutations specifically alter enzyme activity in these sites. We found that 25% of PAD proteins are post-translationally modified by known enzymes that are also targetable with approved drugs according to the DrugBank database [46]. In such cases, pharmacological targeting with known drugs may modulate the aberrant interaction between the upstream enzyme and the substrate protein with PTM-specific disease substitutions. We summarized this as a network of 413 candidate interactions between 47 drugs and 110 diseases where interactions are mediated by PTM enzymes and site-specific substitutions in their substrates encoded by disease genes (Fig. 4C). Systematic queries of drug-disease pairs in the Europe PubMed Central literature database revealed no publications for 79% of pairs (9% with >10 PMIDs), suggesting that most predicted interactions represent novel hypotheses potentially useful for drug repurposing screens. Thus, incorporation of PTM information can help identify information about potentially targetable mechanisms of genetic variant function.

## Discussion

The general and independent signal of mutational constraint in PTM regions establishes these as important factors to consider in variant interpretation. Abundant mutations of monogenic and complex inherited disease as well as cancer [19] emphasize the extent of pathogenic rewiring of PTM-mediated cellular interaction networks. PTM-specific constraint is distinct across the sequence conservation spectrum of human genes, and PTM regions are particularly enriched in disordered sequence that is generally less constrained. Signaling networks are thought to evolve through rapid PTM turnover in clusters such that sequence positions of individual PTM residues are often not conserved [47, 48]. This suggests a model where mutations in PTM sites would be functionally masked by compensation from adjacent sites, however our data indicate that PTM clusters are relatively less tolerant to variation in the population and thus highly functional. Negative selection of PTM regions is also apparent in the sequence sites diverged between human and chimp, highlighting their importance in recent evolution. Therefore, variant function prediction tools are underpowered to evaluate PTM sites solely based on conservation. PTM data integration will improve predictions and provide mechanistic hypotheses.

Integrated statistical modeling of population variation shows that PTM regions are significant predictors of rare substitutions regardless of several well-recognized determinants of variation. We tested six confounding factors with major impact on variation and included all potential interactions to account for complex correlations. The list of confounders is not final and other relevant factors should be further studied. For instance, chromatin state correlates with regional mutation rates in cancer cells [49], and coding sequence variation is impacted by transcription factor binding sites in exonic DNA [50]. Future studies of population variation need to consider variable chromatin state and gene expression in tissue context. Integrated variation analysis of PTM regions, transcription factor binding sites, tissue-specific gene expression, and chromatin state may improve our understanding of the co-evolution of transcriptional and post-translational networks.

Here we focused on a restricted set of post-translational modifications for which most experimental data are available for human proteins, however more than 400 post-translational modifications are known [51]. The proteomics community is mapping PTM sites across a wide range of organisms and disease states [52], thus we expect substantial growth in this area. For example, protein glycosylation is a wide-spread modification with implications in neurological and developmental deficiencies [53], and large-scale experimental data for human proteins are emerging. Further, whole-genome sequencing creates opportunities to evaluate variation of non-coding regulatory elements [54]. Incorporation of functional site-specific information to analyze genome variation can thus help improve associations to phenotype and decipher genetic disease.

## Methods

We provide three collections of PTM-affecting DNA variants of the human genome (hg19) as supplementary information: all possible protein-coding variants, variants seen in the ESP6500 project, and variants seen in the 1000 Genomes project. These datasets are available in S1–S2 Files.

### Post-translational modifications (PTMs)

Experimentally derived post-translational modification (PTM) sites were retrieved from three proteomics databases (PhosphositePlus [13], HPRD [11], PhosphoELM [12]) as 15-mer peptides and matched to longest isoforms of 18,671 completed human RefSeq genes (hg19) allowing multiple matches per sequence, similarly to our earlier analysis [28]. Four modification types with most sites in human proteins were studied (phosphorylation, ubiquitination, acetylation, methylation). Gene and protein IDs were translated to HGNC symbols with g:Profiler [55] software. Disordered and structured protein sequence regions were predicted with DISOPRED2 software [16] version 2.4 using default parameters. PTM regions were defined with seven amino acids of sequence flanking both sides of post-translationally modified protein residue (PTM site). Partially overlapping regions with multiple adjacent PTM residues were merged.

### Genome variation data

Human genome variation data were retrieved as chromosomal nucleotide annotations from online resources. Only missense single nucleotide variants (SNVs) were used while stop codon mutations, small indels, and structural variations were discarded. Protein-level annotations were also discarded from original datasets. Allele frequencies of the Exome Sequencing Project [1] (ESP6500) for 6,503 individuals were downloaded from the Exome Variant Server. Allele frequencies of the 1000 Genomes Project [2] (G1000, Phase 1, Release v3) for 1,092 individuals were downloaded for all Ensembl Gene (ENSG) coordinates from remote VCF files using the Tabix software [56]. We retrieved Derived Allele Frequencies (DAF) relative to the reference human genome to ensure compatibility with our mapping of PTM sites in protein isoforms. Variants with DAF = 0 were removed. Human disease mutations were collected as chromosomal nucleotide annotations from the Human Gene Mutation Database[18] (HGMD) after removing variants with dubious disease association (“DM?”).

Single nucleotide variants (SNVs) from population genome sequencing projects and the HGMD database were mapped to substitutions in human proteins (hg19) using the Annovar [57] software. Non-synonymous variants affecting the same codon were filtered due to ambiguous interpretation of allele frequencies at the protein level. We used a non-redundant set of substitutions by retaining only the longest isoform of each protein. We compared our annotations of protein substitutions with publicly available annotations of the ESP6500 dataset and found and agreement of 97.7%. ActiveDriver software [19] was used to analyze PTM-related substitutions.

Functional impact predictions of substitutions of the ESP6500 dataset and the HGMD database were retrieved from five tools (PhyloP [22], SIFT [23], PolyPhen2 [24], LRT [25], MutationTaster [26]) through the Annovar annotation pipeline, using the cutoff criteria as defined in the dbNSFP database of human non-synonymous SNPs [58]. Functional predictions from the CADD software [27] were retrieved from its website using the Tabix software [56] and classified according to recommended thresholds (score<15 for benign; score≥15 for deleterious).

### Global variation of PTM regions

First we evaluated global distribution of substitutions in PTM regions (modified site ±7 amino acids) relative to substitutions in matched non-PTM protein sequence using two metrics of evolutionary selection: a) proportion of rare substitutions in all substitutions, b) ratio of non-synonymous variants per non-synonymous site to synonymous variants per synonymous site (K_a_/K_s_). We found that proteins with one or more PTM sites are significantly less variable than proteins without any PTM sites, and thus we filtered all non-PTM proteins from all further analyses to avoid systematic biases. The two selection metrics were computed separately for the ESP6500 and G1000 datasets and evaluated with paired one-sided non-parametric tests (Wilcoxon signed rank tests) to estimate statistical significance. To account for variation relative to tolerance to mutations characteristic to different protein groups, we binned all proteins into 100 non-overlapping sets with matched variation such that each set represented one percentile of proteins with similar mean substitution rate per protein sequence length. The paired tests compared PTM-associated substitutions to non-PTM substitutions across the 100 protein sets. For proportion of rare substitutions, Derived Allele Frequency (DAF) cutoff DAF≤0.5% was used to define rare substitutions. The K_a_/K_s_ ratio was computed by accounting for all possible synonymous and non-synonymous sites in protein sequence.

To further validate the three global trends of variation in PTM regions, we repeated the analyses with different subsets of genes, PTM sites and variants. To confirm that the observed PTM constraint is also apparent in the entire proteome, we replicated the analysis on all proteins. We also validated negative selection in more stringent collections of PTM sites by only retaining sites and proteins that were seen in several independent proteomics datasets (2+; 3+; 4+ datasets). To validate robustness of our observations relative to definition of rare substitutions, we tested different DAF values (single allele, two alleles, 1%, 2% of DAF). To check that our observations are not biased by highly expressed proteins that are easier to capture in mass spectrometry, we binned proteins according to median gene expression value in the Human Tissue Expression Atlas of >5,000 microarrays [14]. We also computed the two statistics separately for populations of African and European ancestry of the ESP6500 dataset. We also separately studied the subset of ∼3.5% protein residues diverged between human and chimp from the 100 vertebrate protein alignments of the UCSC Genome Browser [15].

### Disease mutations in PTM regions

Enrichment of disease mutations in PTM regions was evaluated with two strategies. First, we computed the significance of any disease annotations in protein residues in PTM regions with Fisher’s exact tests. As many protein residues are associated to multiple disease annotations and/or substituted residues, we also conducted Poisson exact tests on the total number of disease annotations in PTM regions. Expected values were sampled from the corresponding distributions and shown with ±1 s.d. This analysis was carried out separately for structured and disordered protein sequence due to different variation rates and ascertainment bias of functional predictions. To further study disease mutations of central post-translationally modified residues, we carried out Fisher’s exact tests on each PTM type separately, restricting the background protein sequence to matched types of amino acids to avoid codon bias (S,T,Y for phosphorylation; K for ubiquitination and acetylation; K,R for methylation). The latter analysis was restricted to proteins with specific types of PTMs.

To further investigate the functional prediction bias of disease mutations in PTM regions and disordered sequence, we confirmed that disordered regions are less conserved in ancient as well as recent genes (exclusive sets of genes conserved up to *S. cerevisiae, D. melanogaster, D. rerio, G. gallus, M. musculus, P. troglodytes* retrieved from the Ensembl database [59]). We measured the proportion of deleterious and benign variants predicted by SIFT [23], PolyPhen2 [24] and CADD [27] in structured and disordered regions in both disease variants (HGMD) and population variants (ESP6500) and estimated the significance of under-representation of deleterious variants in disordered regions with Fisher’s exact tests.

### Integrated modeling of PTM variation

Next we confirmed that observations of negative selection in PTM regions are not confounded by other factors in the ESP6500 and the G1000 datasets. We fitted binomial logistic regression models to test the contribution of PTM regions to rare substitutions relative to all substitutions in the presence of six potentially confounding factors contributing to coding genome variation: a) GC content, b) codon usage, c) average sequencing depth (ESP6500 only); d) recombination rate; e) sequence conservation; f) protein disorder. Our null model contained all substitutions as samples, the substitution class as response variable (1 as rare, 0 as common; according to DAF), and as predictive variables the confounding factors and all possible binary and higher-order interactions to account for complex correlations between variables. Our alternative model additionally contained the binary PTM variable and its potential interactions, indicating substitutions in PTM regions. The alternative model was further challenged with a backwards step selection procedure that discarded uninformative predictor variables. The statistical significance of PTM regions in contributing to variation patterns was assessed with an ANOVA procedure with a chi-square test, in which the difference in fits of the null and alternative model was quantified by log likelihood (deviance) and compared relative to change in model complexity (degrees of freedom). The relative contribution of other factors and interactions was also assessed with chi-square tests. Effect directions were estimated from signs of corresponding coefficients.

Confounding factors were defined as follows. GC content was retrieved for every sample (substitution or protein residue) as the percentage of GC nucleotides in the genomic window of 35bp around the SNV (variant location ±17bp). Codon structure was coded as number of nucleotide triplets corresponding to a given amino acid. Average sequence read depth per substitution was retrieved for the ESP6500 dataset while no corresponding values were available for G1000. Recombination rates from the 1000 Genome Project computed by IMPUTE2 software [17] (Phase1 integrated, v3) were matched to every substituted protein residue by retrieving the rate of closest locus with measured recombination rate. Sequence conservation was computed from the 100 vertebrate protein alignments of the UCSC Genome Browser [15] and scored with the BLOSUM62 scores of amino acid substitution (gaps were scored with −10 as used by the BLAST website [60]). Disordered sequences of proteins were predicted with the DISOPRED software [16].

### Evaluating PTM variation in biological context

Having established the global significance of PTM-related variation relative to all coding sequence, we studied PTM-related variation in different contexts including tissue-specific expression, biological processes, and pathways. To account for different mutation rates of structured and disordered protein sequences, we implemented a statistical test based on logistic regression models where the null model classified rare and common variants with disorder as a binary confounding variable, and the alternative model included an additional binary term for PTM regions. Log likelihood ratio test with chi-square statistic was used to compare the alternative and null models, and p-values were corrected with Benjamini-Hochberg False Discovery Rate (FDR).

Groups of highly expressed tissue-specific proteins originate from the Human Protein Atlas [31]. Tissues with numbered subsets were merged (skin, uterus, stomach, soft tissue). An additional group of ubiquitous proteins was defined to include proteins with high expression in 18 or more tissues. This corresponds to robust z-score Z≥2 of tissues per gene. Ubiquitous proteins were removed from tissue-specific categories.

Protein lists corresponding to pathways and processes were retrieved from g:Profiler [55]. We selected biological processes from Gene Ontology [61], pathways from Reactome [62] and KEGG [63], and protein complexes from the CORUM database [64], restricting the analysis to sets with at least five and no more than 1,000 proteins. Pathways were assessed with the permutation-based estimation of rare substitutions and substitution density as described above. Resulting pathways were filtered for significance (FDR *p*<0.05) and subsequently evaluated for enrichment of disease genes using Fisher’s exact test (FDR *p*<0.01) and disease genes with PTM-related substitutions as test set. These pathways and processes were visualized as an enrichment map [65] where shades of light and dark blue represent negative selection, shades of orange and red represent positive selection, node fillings indicate significant selection in the ESP6500 dataset, and node edges show selection in the G1000 dataset. Darker nodes (dark blue, red) are pathways where disease genes with PTM-associated substitutions are enriched. Disease associations of pathways were further explored with word clouds using the R WordCloud package. In word clouds, text sizes correspond to numbers of disease annotations in HGMD that link to PTM-associated substitutions in pathway proteins.

The same strategy was applied to evaluate variation in PTM regions across the spectra of gene expression and evolutionary sequence conservation. Proteins were binned into 100 non-overlapping groups of equal size, based on median expression across 5,000 tissues in the Human Tissue Expression Atlas [14], and median protein residue conservation scores across 100 vertebrates from the UCSC Genome Browser database [15], respectively. Each set was tested with the logistic regression models shown above. Finally, Pearson correlation scores and corresponding p-values were computed between per-bin median expression (conservation) values and relative enrichments/depletions of PTM-related rare substitutions across the 100 bins. Expected values were derived from predicted model responses given estimated model coefficients (mean±standard error of responses). Gene expression analysis was restricted to 9,500 genes encoding PTM proteins that are represented in the microarrays.

### Evaluating PTM variation in biochemical context

Codon structure appeared as an important factor in determining the extent of variation of protein residues, and this was particularly apparent when focusing on single residues such those directly modified by PTMs. To better dissect the biochemical structure of PTM regions and to correctly account for specific variation patterns in particular types of protein residues, we designed an amino acid adjusted permutation strategy. For the PTM regions within a given set of proteins, we computed the observed value as the ratio of rare PTM substitutions over all PTM substitutions. Expected values were derived by 1,000-fold sampling equal numbers of protein residues without replacement, accounting for amino acid frequencies in tested PTM regions or residues.

First, we evaluated variation in central post-translationally modified protein residues as well as proximal (±1–2 amino acids) and distal flanking residues (±3–7 amino acids) using the closest PTM residue as reference. Flanking regions excluded central residues, and wide flanking regions excluded narrow ones. This analysis was performed for all PTMs together and also for different types of PTMs separately. For each comparison, only proteins with the specific type of PTM sites were considered for computing expected values.

Second, we studied variation patterns in clustered PTM sites for the combined set of all PTMs. All protein residues in PTM regions were grouped into five bins based on the number of adjacent PTM sites within ±7 residues (residue adjacent to a single site, two sites, three sites, four sites, five or more sites). Observed and expected substitutions were derived as described above.

### Evaluating variation and disease associations of kinase motifs and motif-breaker sites

Using our previously developed computational strategy MIMP [28] [Wagih, Reimand, Bader, *submitted*], we predicted high-confidence binding sites of 124 kinases using a reliable subset of kinase binding models (position weight matrices) we collected earlier. To increase the confidence of our kinase-substrate network, we only predicted motifs in protein sequence that flanked experimentally verified phosphorylation sites. In brief, a kinase was considered to bind a phosphorylation site if its binding score exceeded the bottom 10% of positive control sequences and was above 90% of negative control sequences sampled from non-phosphorylated sites with central S,T,Y residues. Using this set of predicted kinase sites, we performed all exhaustive mutations of predicted sites and selected residues that would lead to strong loss of binding motif if substituted (≥4-fold reduction of binding score). These residues are referred to as motif-breakers.

Motif-breaker sites were grouped by kinases (corresponding motifs) and analyzed separately for enrichment of rare variants in the ESP6500 dataset. We used the amino acid-weighted permutation strategy shown above to compute expected values of proportions of rare substitutions, where amino acids corresponding to motif-breaker sites were sampled without replacement from all proteins with phosphorylation sites. Kinase-specific motif-breaker sites were also subjected to enrichment analysis of disease mutations with Fisher’s exact tests and resulting p-values were corrected with FDR. The set of 24 kinases with motif-breaker site-specific negative selection, disease mutation enrichment or both signals were selected for further analysis.

Motif-breaker sites of selected kinases were collected and assembled into a network of interactions between kinases and predicted substrate proteins. Disease genes with known PTM mutations from HGMD were highlighted separately. The network was visualized with the Cytoscape software [66]. Network node degree (i.e., number of bound kinases) of disease genes and other genes was assessed with the Wilcoxon test. Amino acid types and positions of all motif-breaker sites relative to central phosphorylated residues were assembled into a summarized position weight matrix and visualized as a logo using the WebLogo software [67].

### Disease genes with mutation enrichment in PTM regions

We used our previously developed ActiveDriver method [19] to evaluate HGMD disease mutations in PTM sites using the entire collection of PTMs. In brief, a Poisson regression model with protein disorder as a confounding factor was used to decide whether a particular PTM site contains more mutations than expected from protein-wide average. Protein-wide significance score was estimated as an aggregate of site-specific p-values, and the results were corrected for multiple testing (results with FDR *p*<0.05 were selected as significant genes). The number of independent records per amino acid position in the HGMD database was used as proxy of mutation frequency, reflecting different underlying diseases and certainty in particular disease variants. High-confidence cancer genes were retrieved from earlier review papers [68–71] via the CancerGenes database [38]. Enrichment of cancer genes was conducted with Fisher’s exact test.

### Drug-disease interactions of disease genes with PTM-associated SNVs

Using approved pharmaceutical drugs known to target these enzymes the DrugBank database [46], we constructed a directed network of drug-disease interactions. The network contains hierarchical associations of the following components: a) pharmaceutical compound (drug) acting on a PTM enzyme, b) druggable PTM enzyme binding a disease gene according to experimental evidence from proteomics databases, c) confirmed disease gene with enriched PTM mutations from ActiveDriver analysis, where gene mutations from HGMD specifically affect PTM sites bound by the above PTM enzyme, and d) disease annotations from the HGMD database that associate human diseases to gene mutations that occur in the binding site of the PTM enzyme. We applied ActiveDriver analysis to pre-select disease genes with PTM-specific mutational enrichment to de-convolute the drug-gene network and focus on abundant and PTM-specific disease annotations.

## Supporting Information

S1 File All PTM-associated genome variants (missense SNVs, hg19) as tab-separated text files (archive part A). The compressed ZIP archive includes three tables with chromosomal coordinates, corresponding protein substitutions, and related PTM annotations. Two tables contain variants seen in population genomics projects (ESP6500, 1000 Genomes). The third table contains all potential single nucleotide variants of the human genome that affect PTM sites (chromosomes 1–8). The file also contains three index files for queries with the Tabix software.
(ZIP)

S2 File All PTM-associated genome variants (archive part B). The archive contains a table with all potential SNVs that affect PTM sites (chromosomes 9–22, X, Y). The files are split due to size restrictions. The archive also contains an index file for queries with the Tabix software.
(ZIP)

S1 Fig Distribution of PTM regions per gene. Two thirds of human proteins have at least one PTM region.
(PDF)

S2 Fig Lengths of PTM regions. Half (54%) of PTM regions have one post-translationally modified residue and PTM region of < = 15 amino acids (central site and flanking sequence +/− 7 residues). Regions with less than 15 residues involve PTM sites in protein termini. Regions spanning hundreds of residues involve hyper-phosphorylated sites.
(PDF)

S3 Fig Proteins with PTM sites are less variable (left) and contain more rare variants (right) compared to proteins with no PTM sites. P-values are computed with Wilcoxon test. Rare substitutions comprise variants with derived allele frequency DAF≤0.5% in the ESP6500 dataset.
(PDF)

S4 Fig Negative selection of PTM regions is apparent when considering all proteins with and without PTM sites. Top: Fraction of rare substitutions in PTM regions compared to non-PTM protein sequences. Bottom: ratio of non-synonymous to synonymous variants in PTM regions vs non-PTM protein sequence. P-values are computed using paired Wilcoxon tests across bins representing 1% of proteins with matched variation.
(PDF)

S5 Fig Negative selection of PTM regions is apparent when considering high-confidence PTM sites. PTM sites were filtered based on number of associated publications (xPMID—X or more PubMed IDs). Left: Fraction of rare substitutions in PTM regions compared to non-PTM protein sequences. Right: ratio of non-synonymous to synonymous variants in PTM regions compared to non-PTM protein sequence.
(PDF)

S6 Fig Enrichment of rare variants in PTM regions is apparent when binning genes according to variation in the ESP6500 dataset. Variation is quantified by number of substituted protein residues per total protein sequence. Each bar represents 1% of proteins with similar variation. Y-axis shows log2 ratios of rare variant proportions in PTM regions over proportions in non-PTM protein sequence. Panels show results for all sequence (top), and separately for structured (middle) and disordered sequence (bottom).
(PDF)

S7 Fig Lower K_a_/K_s_ ratio of PTM regions (ratio of non-synonymous to synonymous variants) is apparent when binning genes according to variation in the ESP6500 project. Variation is quantified by number of substituted protein residues per total protein sequence. Each bar represents 1% of proteins with similar variation. Y-axis shows log2 ratios of K_a_/K_s_ ratios in PTM regions over non-PTM protein sequence. Panels represent K_a_/K_s_ ratios in all sequence (top), and separately for structured (middle) and disordered sequence (bottom).
(PDF)

S8 Fig Rare PTM substitutions are more frequent in lowly and highly expressed genes. PTM-related substitutions are compared to substitutions in non-PTM sequence of proteins with matched gene expression, using protein disorder as confounding factor. Each point represents 1% of genes with similar median gene expression intensity across >5,000 microarrays with human tissues (error bars show model predictions with +/−1 standard error). 9,500 genes with PTM sites and gene expression information are studied.
(PDF)

S9 Fig Higher proportion of rare substitutions in PTM regions is apparent when using different cutoff values of Derived Allele Frequency (DAF, %) to define rare substitutions. Boxplots represent rare substitution proportions for 100 groups of genes each representing 1% of genes with matched variation (total number of substitutions per sequence position). P-values are computed with paired (signed-rank) Wilcoxon tests.
(PDF)

S10 Fig Negative selection of PTM regions is apparent when considering African American (n = 2,203) and European American (n = 4,300) populations of the ESP6500 project. Top: fraction of rare substitutions in PTM regions and variation-matched non-PTM regions. Bottom: K_a_/K_s_ ratio in PTM regions relative to matched protein-coding sequence. P-values are computed with paired Wilcoxon tests.
(PDF)

S11 Fig Negative selection of PTM regions is apparent when only considering 375,247 protein residues diverged between human and chimp. All individuals, African American (n = 2,203) and European American (n = 4,300) individuals of the ESP6500 dataset were analysed. Barplots show proportion of rare substitutions in PTM regions (47,559 residues). Expected proportion is obtained by 1,000 permutations and visualised with red boxplots. Proportion of rare PTM substitutions in all protein sequence is shown as control.
(PDF)

S12 Fig In the 1000 Genomes dataset, PTM regions are 4^th^ most important determinant of rare substitutions after conservation, codon bias, GC content. Logistic regression models to classify rare substitutions were fitted with all confounding factors and their interactions using all substitutions as samples. Full model was challenged with backwards selection to remove insignificant predictors. The final model was assessed with analysis of deviance. PTM region p-value indicates log-likelihood ratio test between null model (all confounding factors predict rare substitutions) and alternative model (additional terms for PTM regions and interactions, subject to backwards selection). Signs of regression coefficients show direction of effect.
(PDF)

S13 Fig Rare PTM substitutions are more frequent in proteins with high and low evolutionary sequence conservation. PTM-related substitutions are compared to substitutions in non-PTM sequence of conservation-matched proteins with protein disorder as confounding factor. Each bar represents 1% of genes with similar median conservation across 100 vertebrates (error bars show model predictions with +/−1 standard error).
(PDF)

S14 Fig PTM regions are enriched in substitutions of human disease mutations of the HGMD database. Figure shows observed and expected values for all disease annotations such that substitutions with multiple annotations are accounted for. Only proteins with at least one PTM site are studied. Expected values +/−1 standard deviation and significance p-values are computed from the Poisson distribution. Modified residues (DI), proximal (±2 residues) and distal flanking regions (±7 residues) are shown.
(PDF)

S15 Fig Central residues of PTMs are enriched in HGMD disease substitutions when only amino acids corresponding to modification type are considered as background. Phosphorylation (S,T,Y residues), ubiquitination (K), acetylation (K), and methylation (K,R) are shown separately, and proteins with respective PTM sites are used as background. Expected values (±1 s.d.) and mutational enrichments are computed from the binomial distribution.
(PDF)

S16 Fig Disordered protein sequence is less conserved than structured protein sequence across ancient and recent human genes. Panels represent genes conserved in human and denoted species.
(PDF)

S17 Fig Disordered protein sequence is enriched in PTM regions. P-value is computed with Fisher’s exact test.
(PDF)

S18 Fig Population variants (ESP6500) and disease mutations (HGMD) in disordered regions are less often considered deleterious by variant function prediction tools such as PolyPhen2, SIFT, and CADD. Disordered protein sequence is less conserved and this affects variant function prediction that largely relies on evolutionary conservation. P-values are computed with Fisher’s exact test.
(PDF)

S19 Fig Quantification of proteins, PTM region sizes and PTM-related substitutions (ESP6500) in tissue-specific proteins with significant PTM-specific selection. (PDF)

S20 Fig Distribution of pathways and processes with significant positive and negative selection in PTM regions (FDR *p*<0.05). Selection was measured with logistic regression tests with protein disorder as confounding factor. We tested 9,084 biological processes (GO), pathways (KEGGm Reactome), and protein complexes (CORUM). Enrichment of disease genes with PTM mutations was computed with Fisher’s exact test (FDR *p*<0.01).
(PDF)

S21 Fig Number of modified proteins, PTM region sizes and PTM-related substitutions (ESP6500) in pathways and processes with significant PTM-specific selection. (PDF)

S22 Fig Number of modified proteins, PTM region sizes and PTM-related substitutions (ESP6500) grouped by PTM type and proximity to central site (DI, direct subsitution of PTM site; N1, 1–2 residues from PTM site; N2, 3–7 residues). (PDF)

S23 Fig Number of PTM sites (modified residues) per PTM region. 46% of PTM regions have more than one site.
(PDF)

S24 Fig Number of modified proteins, region sizes and PTM-related substitutions (ESP6500) in regions with multiple adjacent modified residues. (PDF)

S25 Fig Word clouds show human diseases (top) and disease genes (bottom) with mutations in kinase motif-breaker sites. Letter size and color indicates frequency of mutation annotation in the HGMD database. Clouds are not drawn to scale.
(PDF)

S26 Fig Number of modified proteins, motif-breaker sites and substitutions (ESP6500) of kinases with significant disease mutation enrichment and/or population constraint in motif-breaker sites. (PDF)

S27 Fig Kinase motifs and motif-breaker sites with negative selection and/or disease mutation enrichment (24 kinases). Protein residues in motif-breaker sites are highlighted with red boxes.
(PDF)

S28 Fig HGMD disease annotations corresponding to 152 genes enriched in PTM-related mutations highlighed by ActiveDriver (PAD genes, FDR *p*<0.05). Letter size and color indicates number of disease annotations.
(PDF)
