# Supplementary figures and images for: Evolutionary Constraint and Disease Associations of Post-Translational Modification Sites in Human Genomes
Source: PLoS Genet. 2015 Jan 22;11(1):e1004919. doi: 10.1371/journal.pgen.1004919 (PMC4303425; doi:10.1371/journal.pgen.1004919)

PTM regions per gene  
12364 genes with 1+ PTM

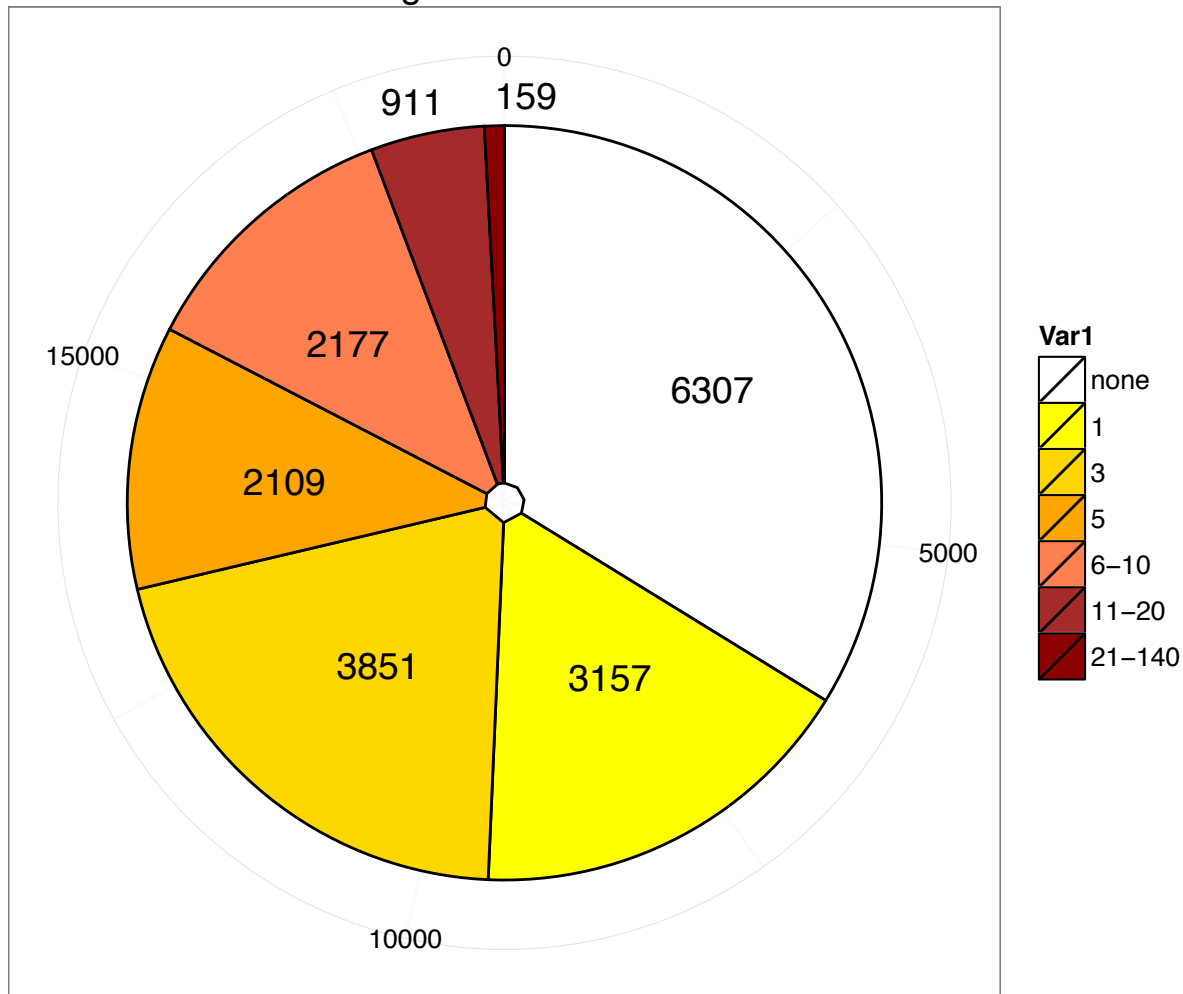

Supplement: S1 Fig — Two thirds of human proteins have at least one PTM region. (PDF) [file pgen.1004919.s003.pdf]

Length distribution of PTM elements (total n=55543)

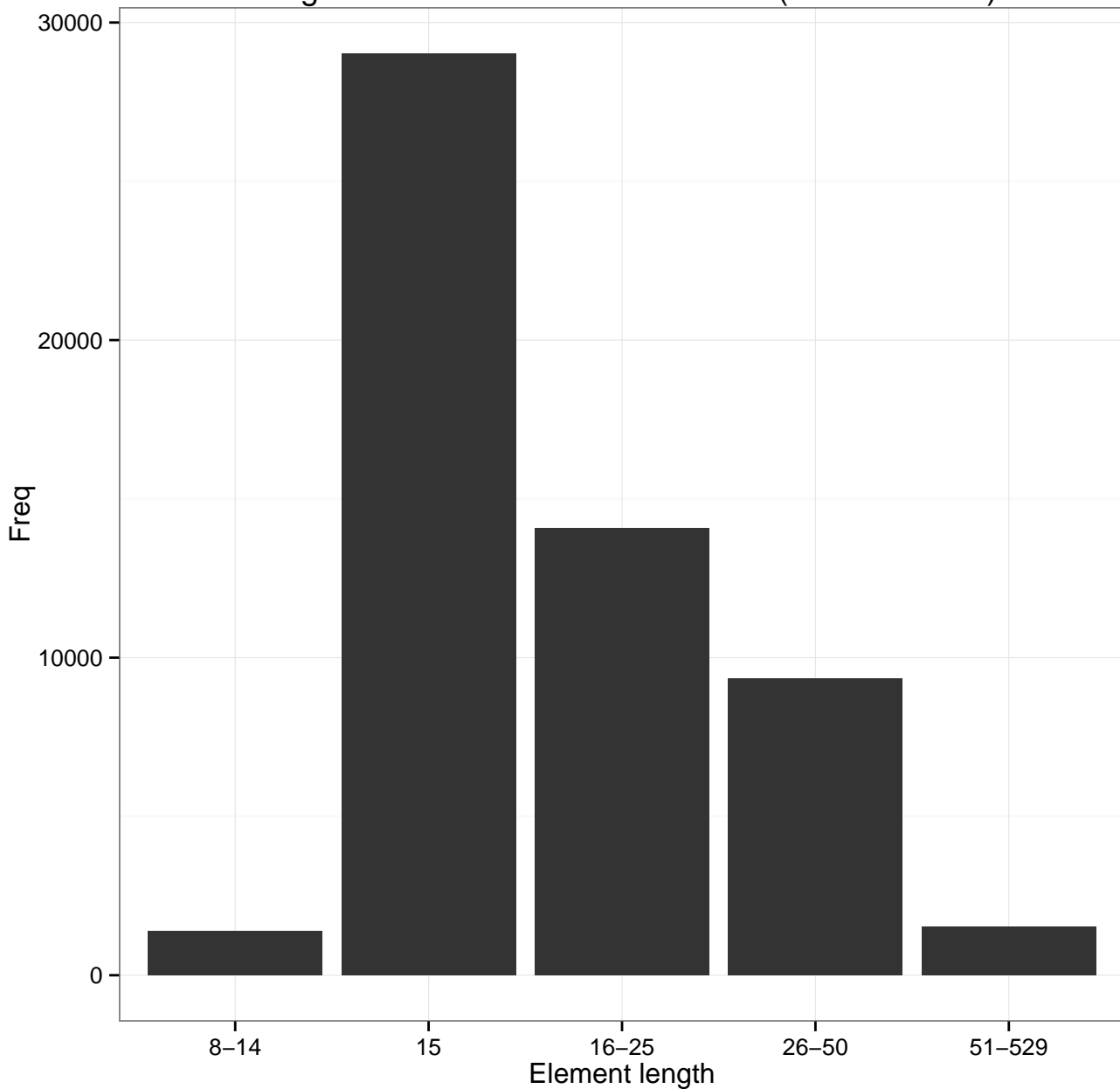

Supplement: S2 Fig — Half (54%) of PTM regions have one post-translationally modified residue and PTM region of < = 15 amino acids (central site and flanking sequence +/− 7 residues). Regions with less than 15 residues involve PTM sites in protein termini. Regions spanning hundreds of residues involve hyper-phosphorylated sites. (PDF) [file pgen.1004919.s004.pdf]

Proteins with PTM sites  
are less variable

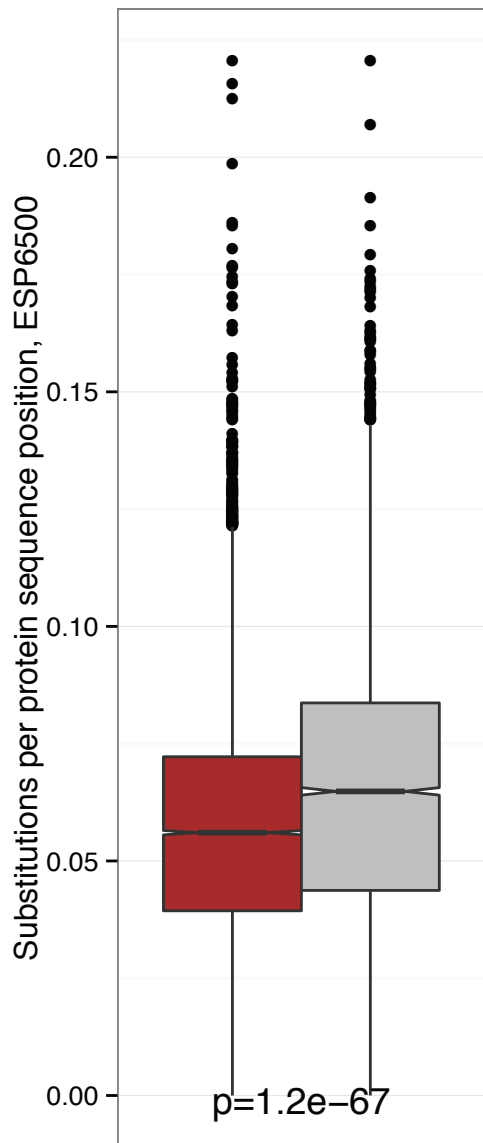

Proteins with PTM sites  
have more rare variants

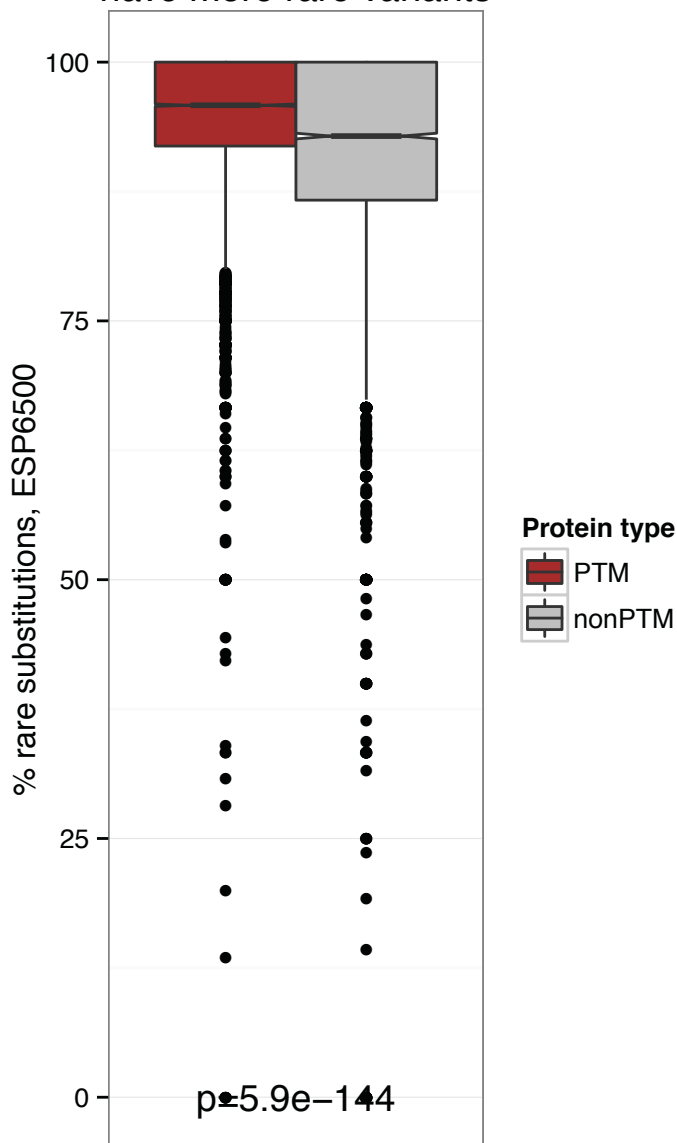

Supplement: S3 Fig — P-values are computed with Wilcoxon test. Rare substitutions comprise variants with derived allele frequency DAF≤0.5% in the ESP6500 dataset. (PDF) [file pgen.1004919.s005.pdf]

# PTM regions have excess rare variants (DAF $\leq 0.5$ ) across the variation spectrum

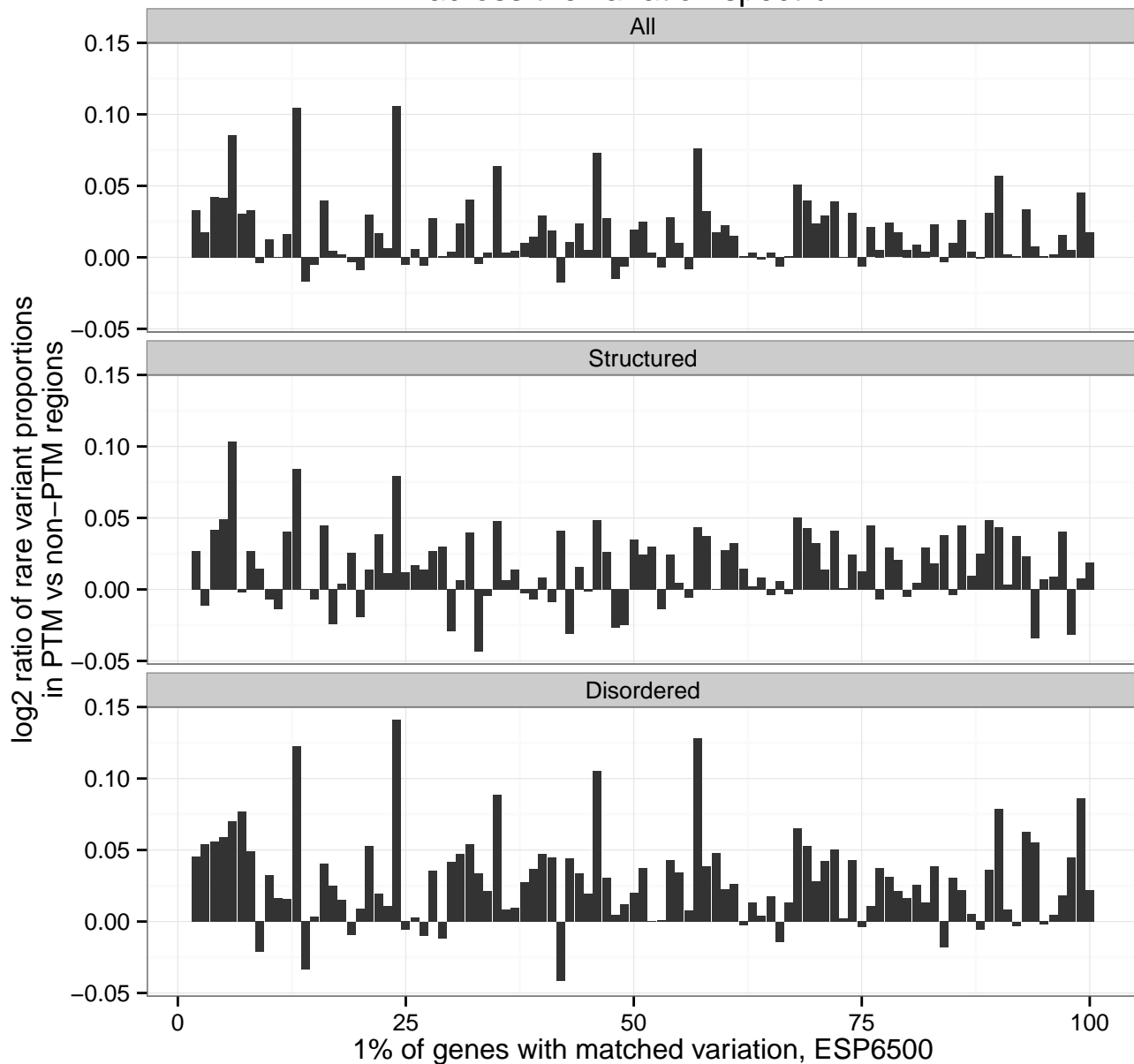

Supplement: S6 Fig — Variation is quantified by number of substituted protein residues per total protein sequence. Each bar represents 1% of proteins with similar variation. Y-axis shows log2 ratios of rare variant proportions in PTM regions over proportions in non-PTM protein sequence. Panels show results for all sequence (top), and separately for structured (middle) and disordered sequence (bottom). (PDF) [file pgen.1004919.s008.pdf]

Rare PTM SNVs are enriched across the expression intensity spectrum  
Pearson  $r=0.12$ ;  $p=0.25$

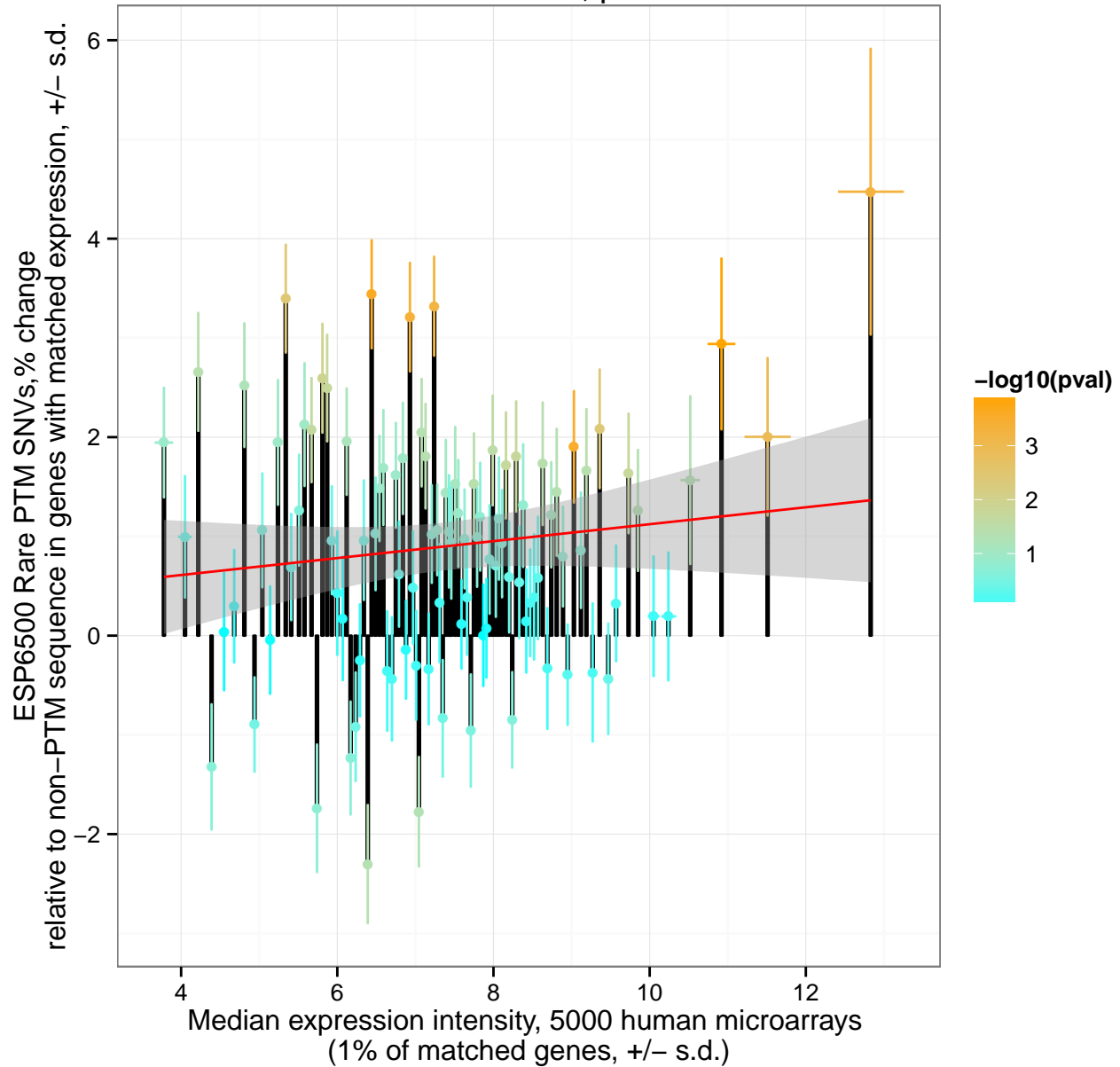

Supplement: S8 Fig — PTM-related substitutions are compared to substitutions in non-PTM sequence of proteins with matched gene expression, using protein disorder as confounding factor. Each point represents 1% of genes with similar median gene expression intensity across >5,000 microarrays with human tissues (error bars show model predictions with +/−1 standard error). 9,500 genes with PTM sites and gene expression information are studied. (PDF) [file pgen.1004919.s010.pdf]

# Rare substitutions in PTM and non-PTM protein sequence

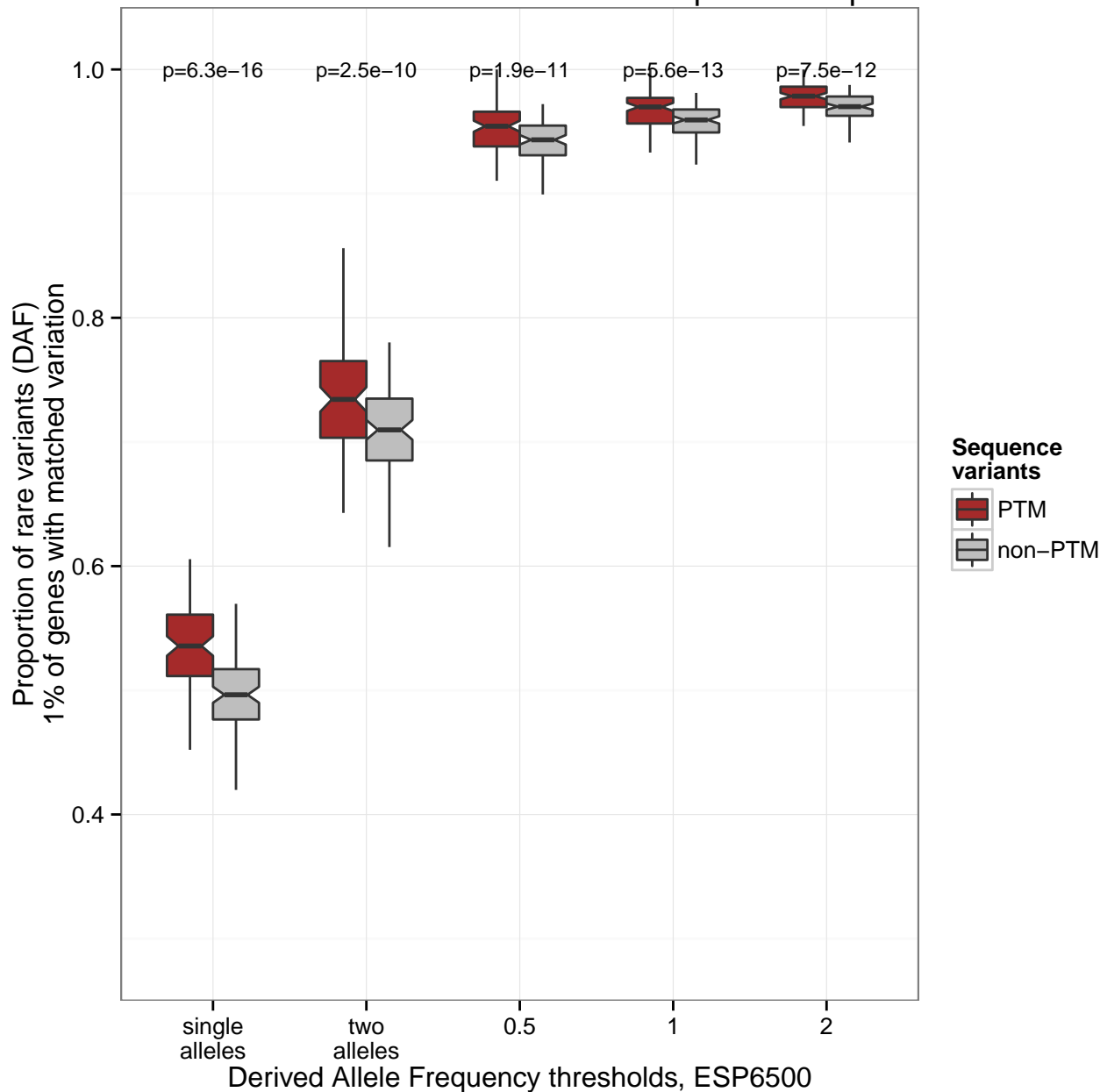

Supplement: S9 Fig — Boxplots represent rare substitution proportions for 100 groups of genes each representing 1% of genes with matched variation (total number of substitutions per sequence position). P-values are computed with paired (signed-rank) Wilcoxon tests. (PDF) [file pgen.1004919.s011.pdf]

# Negative selection of PTM regions in residues with human–chimp divergence

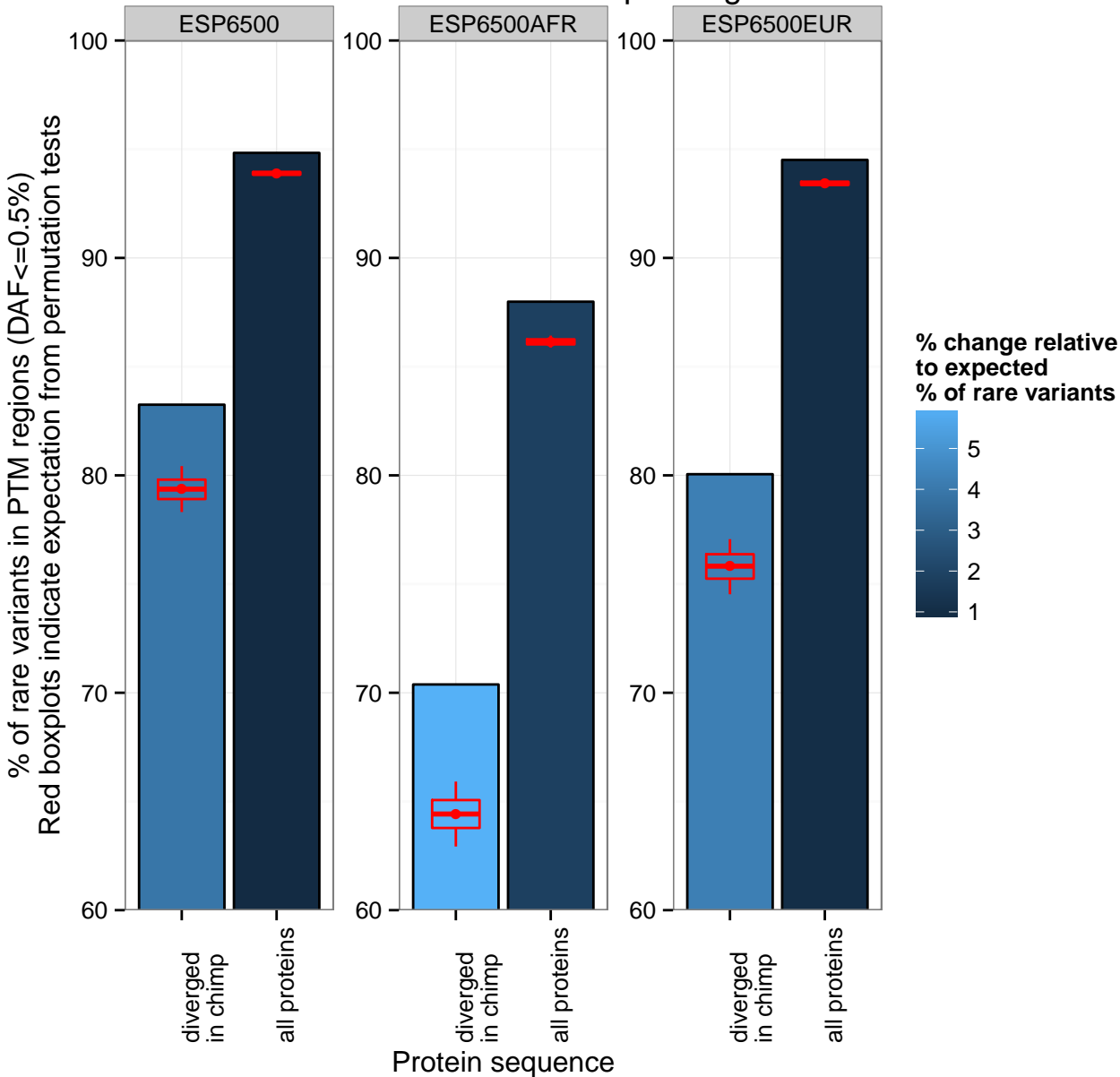

Supplement: S11 Fig — All individuals, African American (n = 2,203) and European American (n = 4,300) individuals of the ESP6500 dataset were analysed. Barplots show proportion of rare substitutions in PTM regions (47,559 residues). Expected proportion is obtained by 1,000 permutations and visualised with red boxplots. Proportion of rare PTM substitutions in all protein sequence is shown as control. (PDF) [file pgen.1004919.s013.pdf]

## Factors contributing to abundance of rare variants in 1000 Genomes

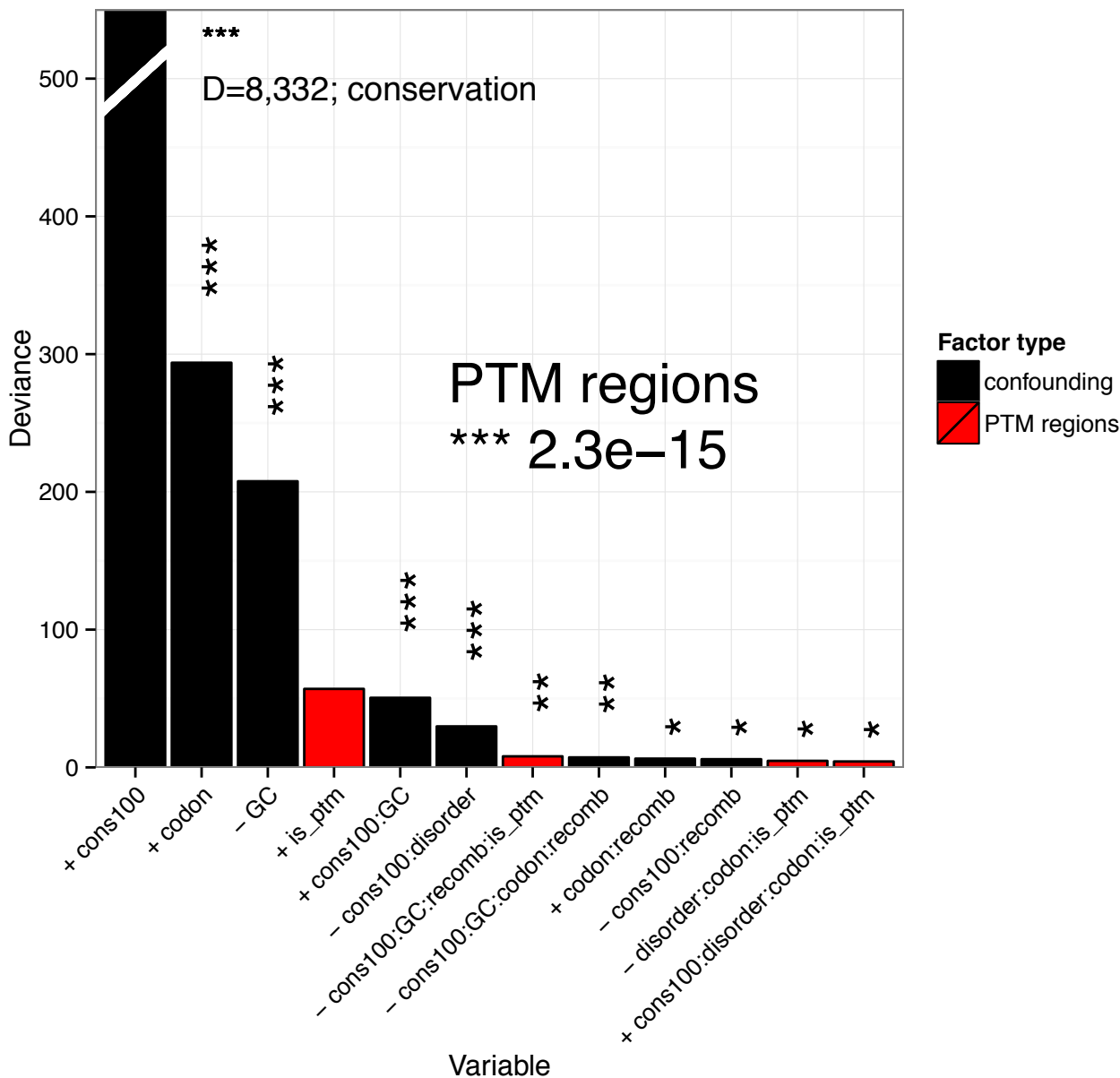

Supplement: S12 Fig — Logistic regression models to classify rare substitutions were fitted with all confounding factors and their interactions using all substitutions as samples. Full model was challenged with backwards selection to remove insignificant predictors. The final model was assessed with analysis of deviance. PTM region p-value indicates log-likelihood ratio test between null model (all confounding factors predict rare substitutions) and alternative model (additional terms for PTM regions and interactions, subject to backwards selection). Signs of regression coefficients show direction of effect. (PDF) [file pgen.1004919.s014.pdf]

# Rare PTM SNVs are enriched across the conservation spectrum

Pearson  $r=-0.088$ ;  $p=0.38$

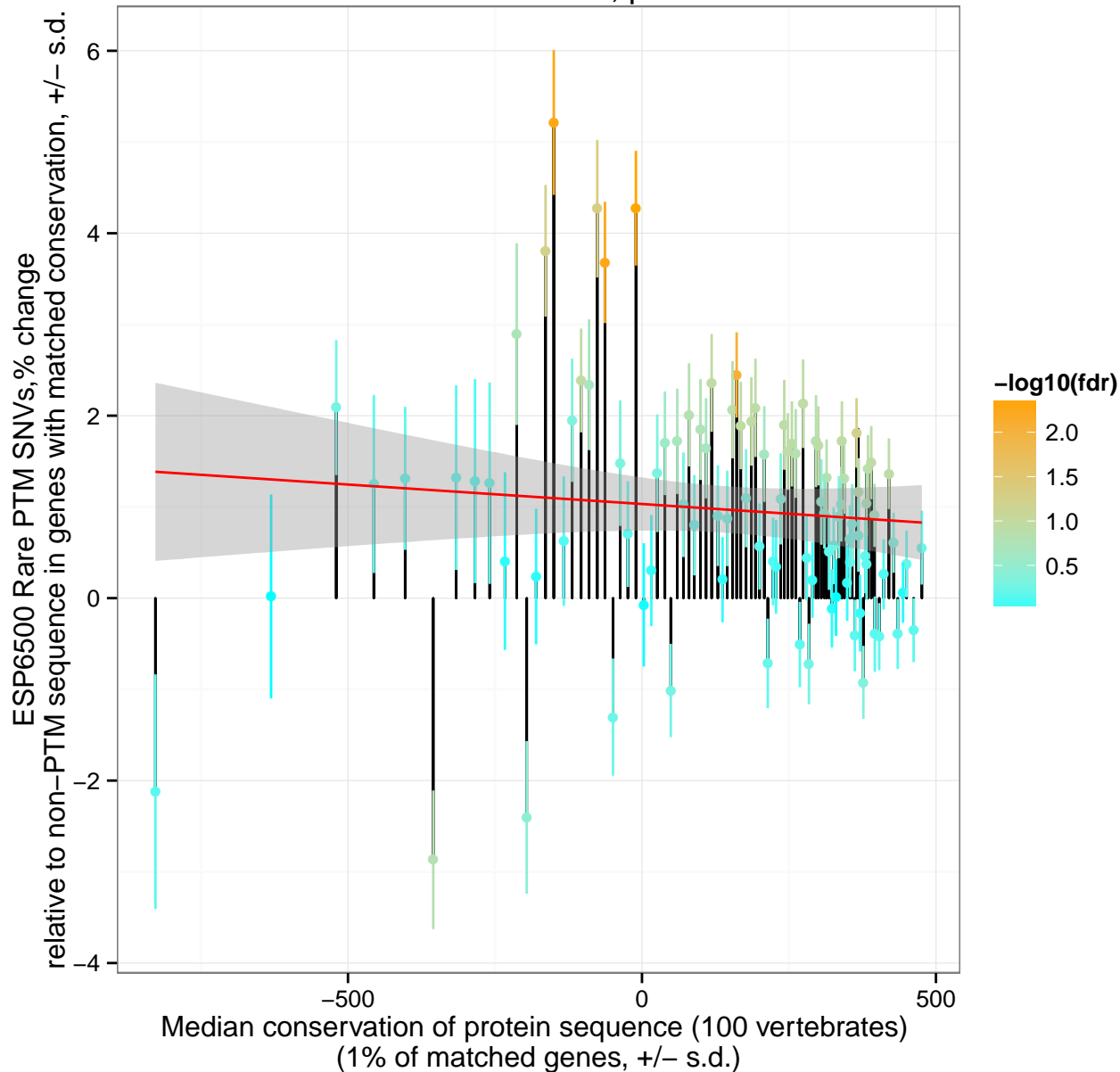

Supplement: S13 Fig — PTM-related substitutions are compared to substitutions in non-PTM sequence of conservation-matched proteins with protein disorder as confounding factor. Each bar represents 1% of genes with similar median conservation across 100 vertebrates (error bars show model predictions with +/−1 standard error). (PDF) [file pgen.1004919.s015.pdf]

# HGMD disease mutations in PTM elements, Poisson test

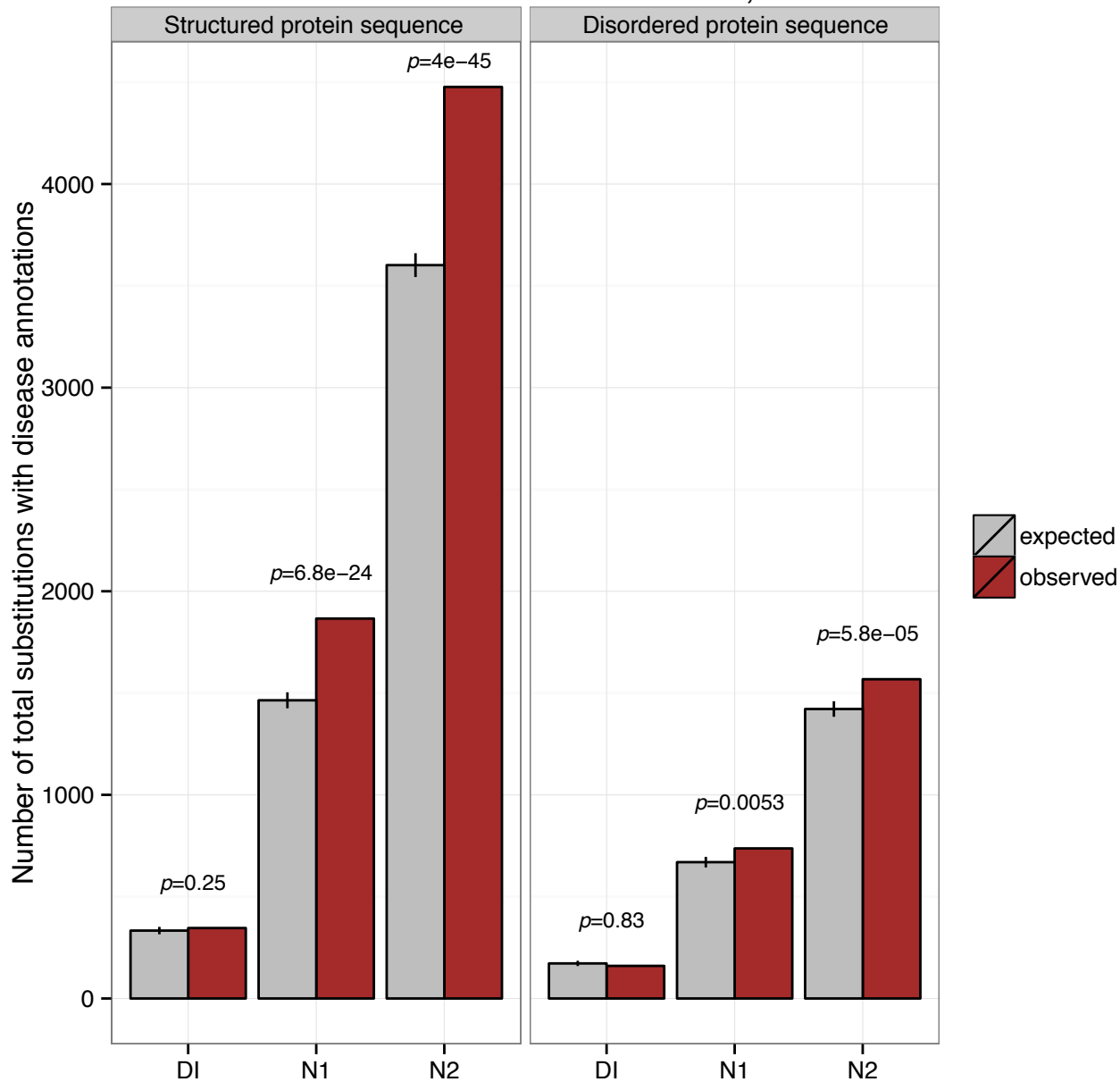

Supplement: S14 Fig — Figure shows observed and expected values for all disease annotations such that substitutions with multiple annotations are accounted for. Only proteins with at least one PTM site are studied. Expected values +/−1 standard deviation and significance p-values are computed from the Poisson distribution. Modified residues (DI), proximal (±2 residues) and distal flanking regions (±7 residues) are shown. (PDF) [file pgen.1004919.s016.pdf]

# HGMD disease mutations in PTM residues, unique mutated residues

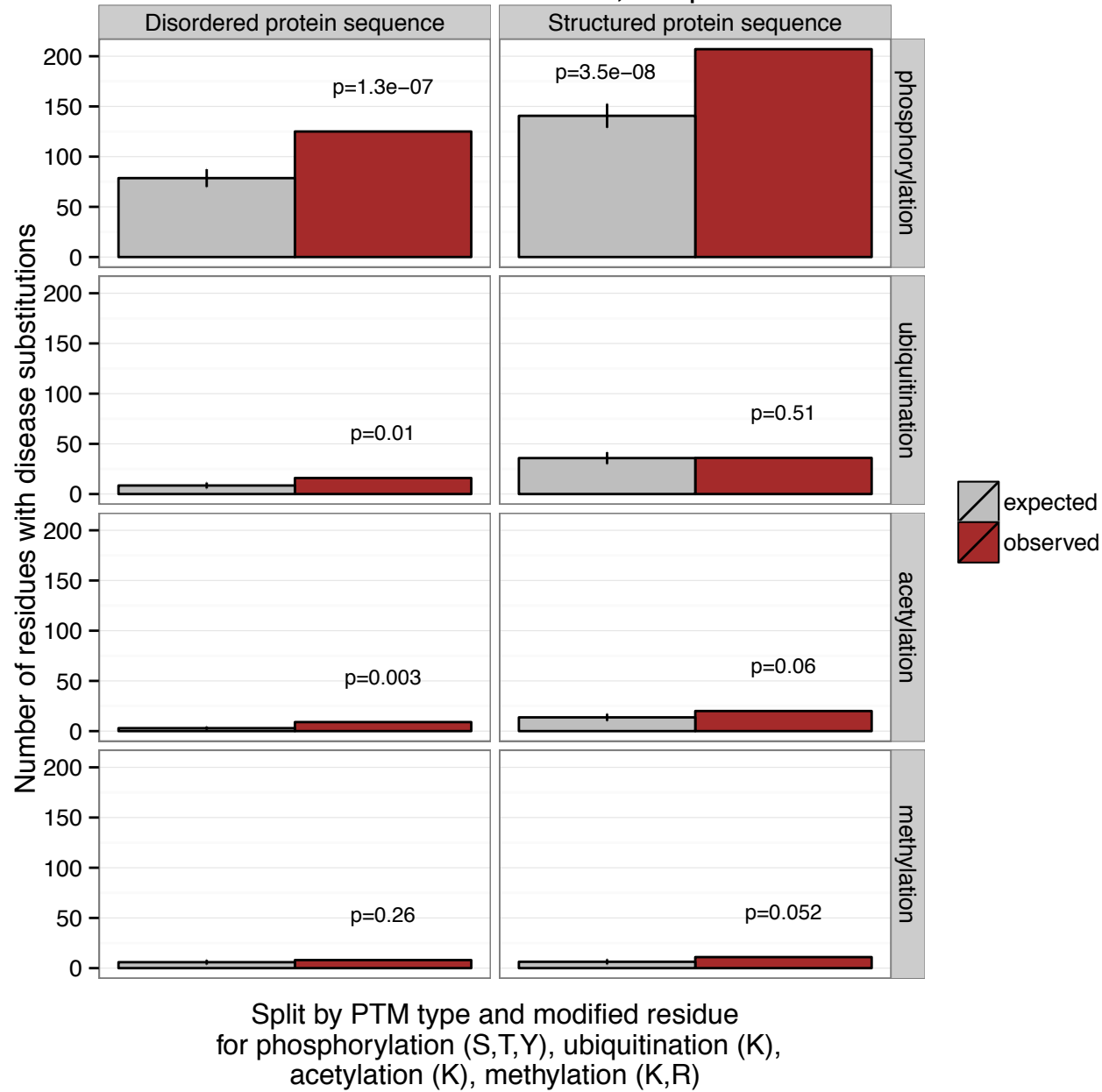

Supplement: S15 Fig — Phosphorylation (S,T,Y residues), ubiquitination (K), acetylation (K), and methylation (K,R) are shown separately, and proteins with respective PTM sites are used as background. Expected values (±1 s.d.) and mutational enrichments are computed from the binomial distribution. (PDF) [file pgen.1004919.s017.pdf]

# Conservation of structured and disordered protein sequence

## Ancient and recent human genes

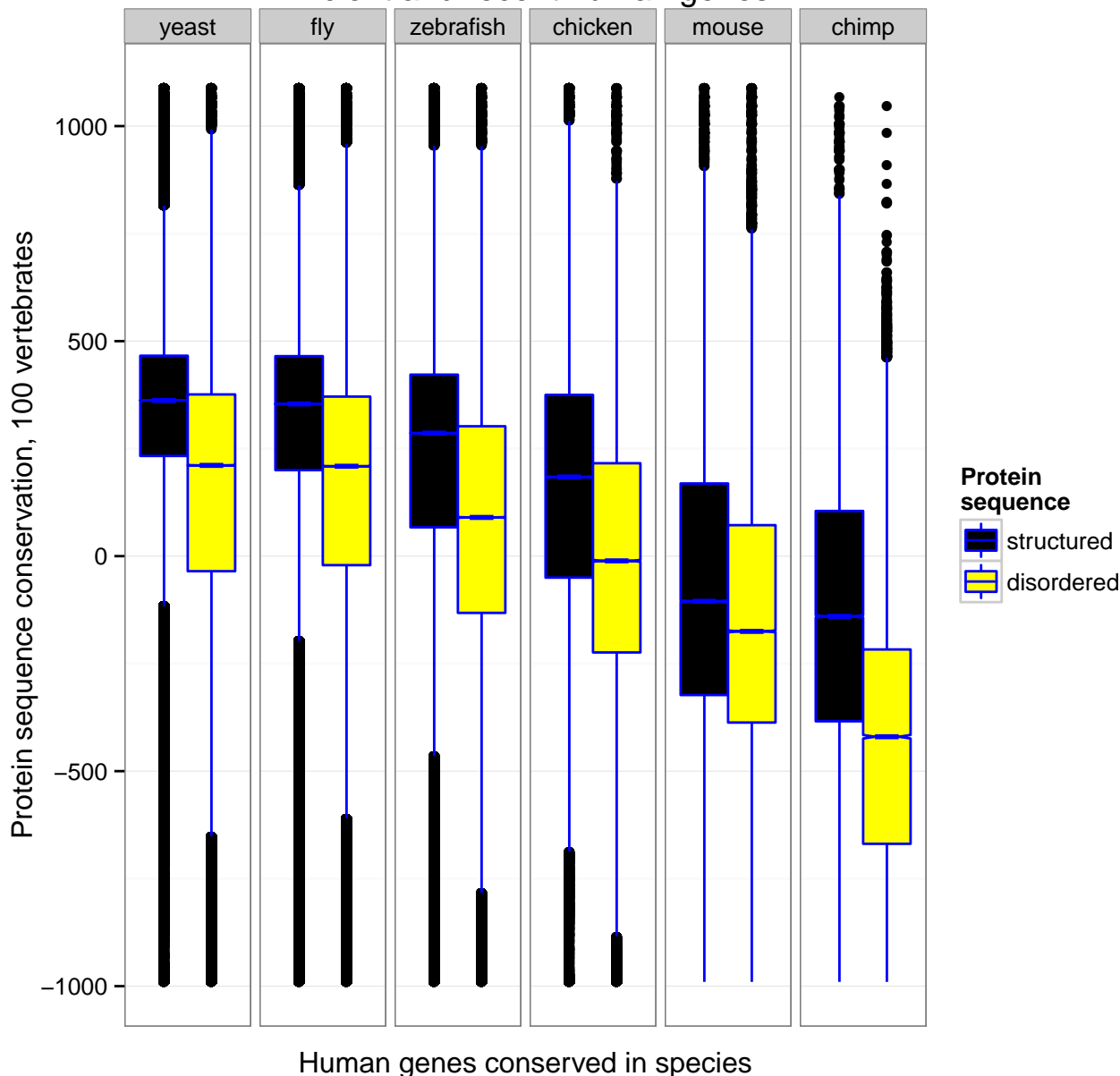

Supplement: S16 Fig — Panels represent genes conserved in human and denoted species. (PDF) [file pgen.1004919.s018.pdf]

# Enrichment of PTM regions in disordered protein sequence

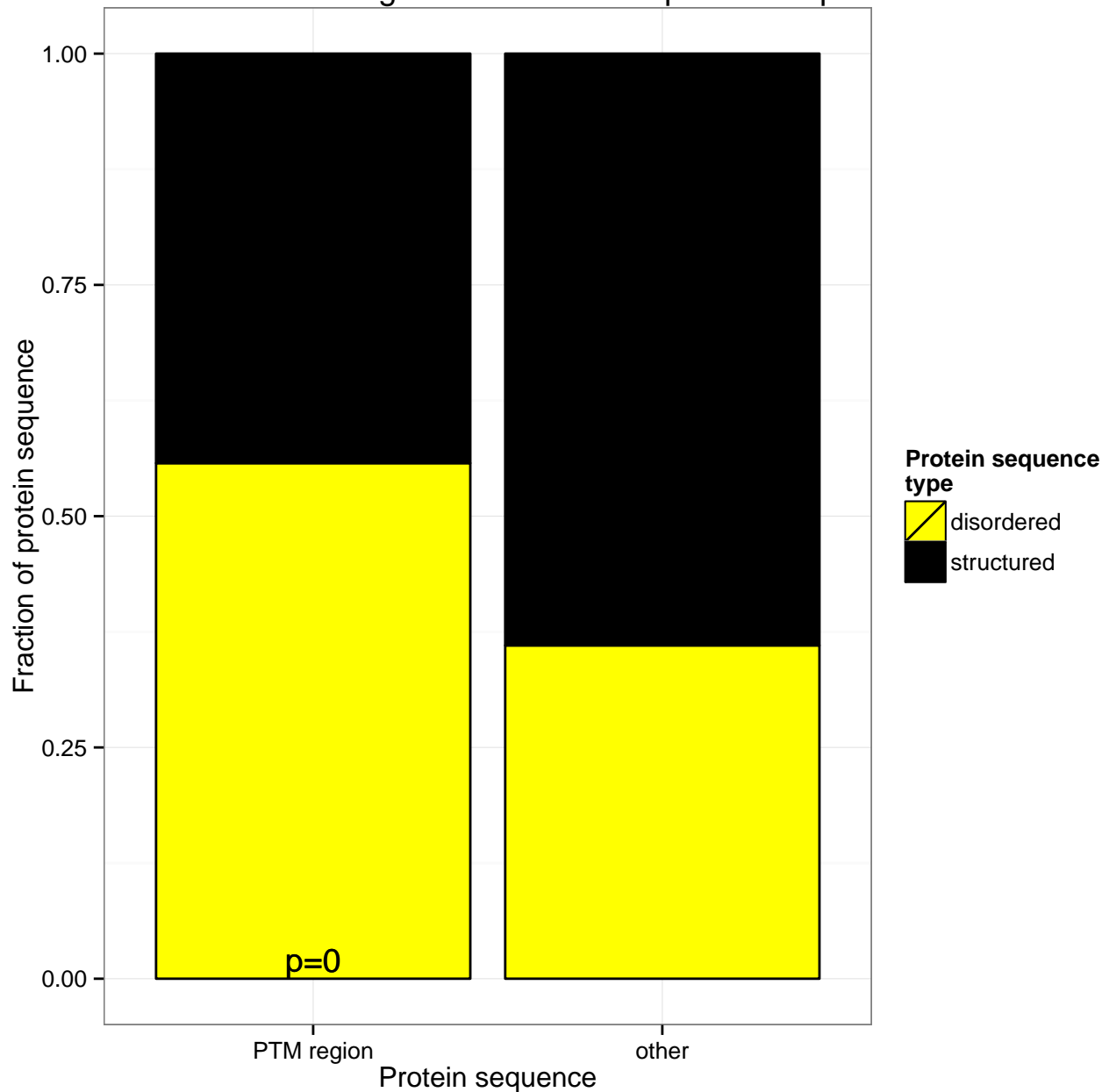

Supplement: S17 Fig — P-value is computed with Fisher’s exact test. (PDF) [file pgen.1004919.s019.pdf]

# Under-representation of variants with deleterious predictions in disordered sequence

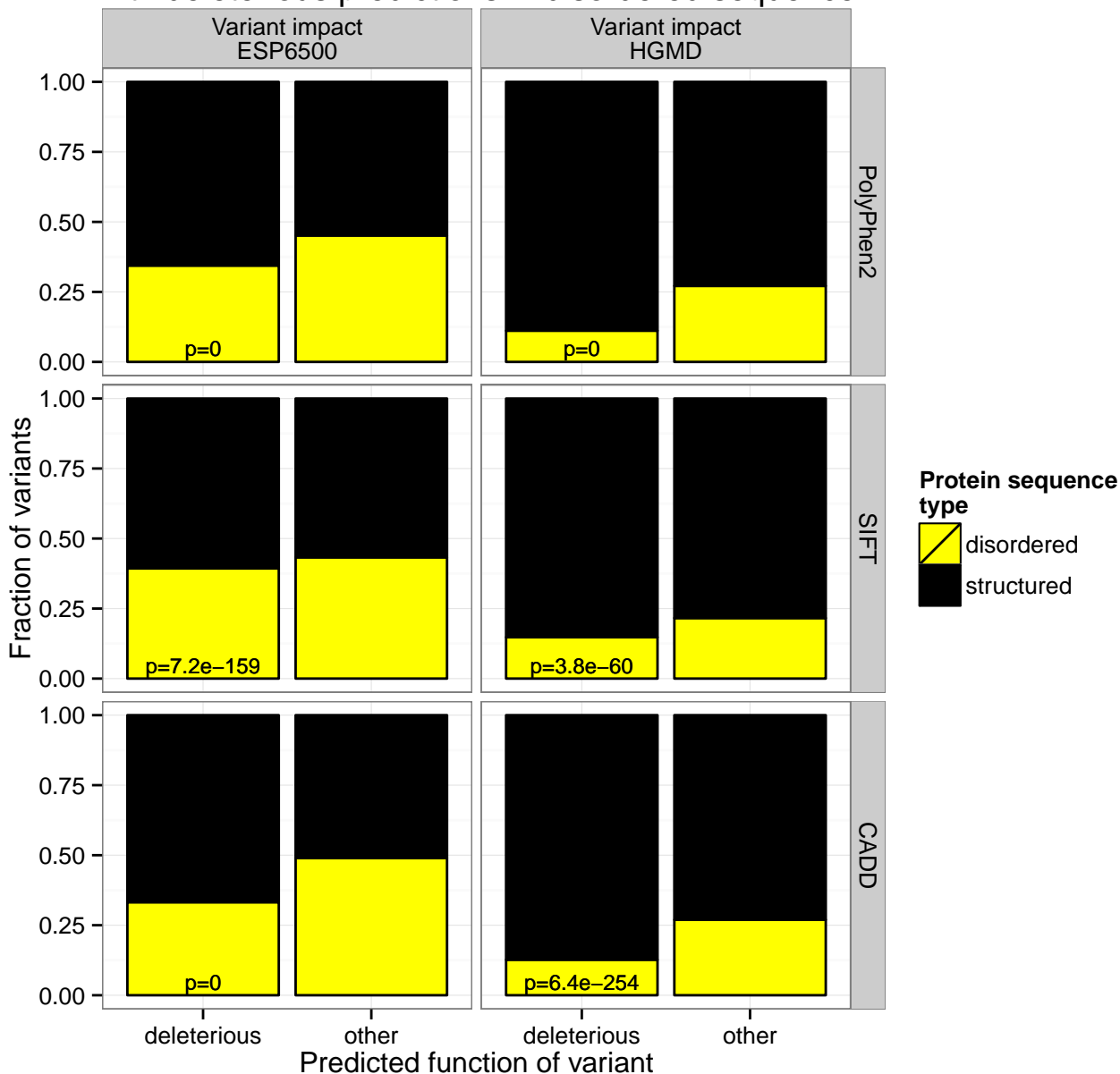

Supplement: S18 Fig — Disordered protein sequence is less conserved and this affects variant function prediction that largely relies on evolutionary conservation. P-values are computed with Fisher’s exact test. (PDF) [file pgen.1004919.s020.pdf]

# PTM statistics of tissue-specific genes

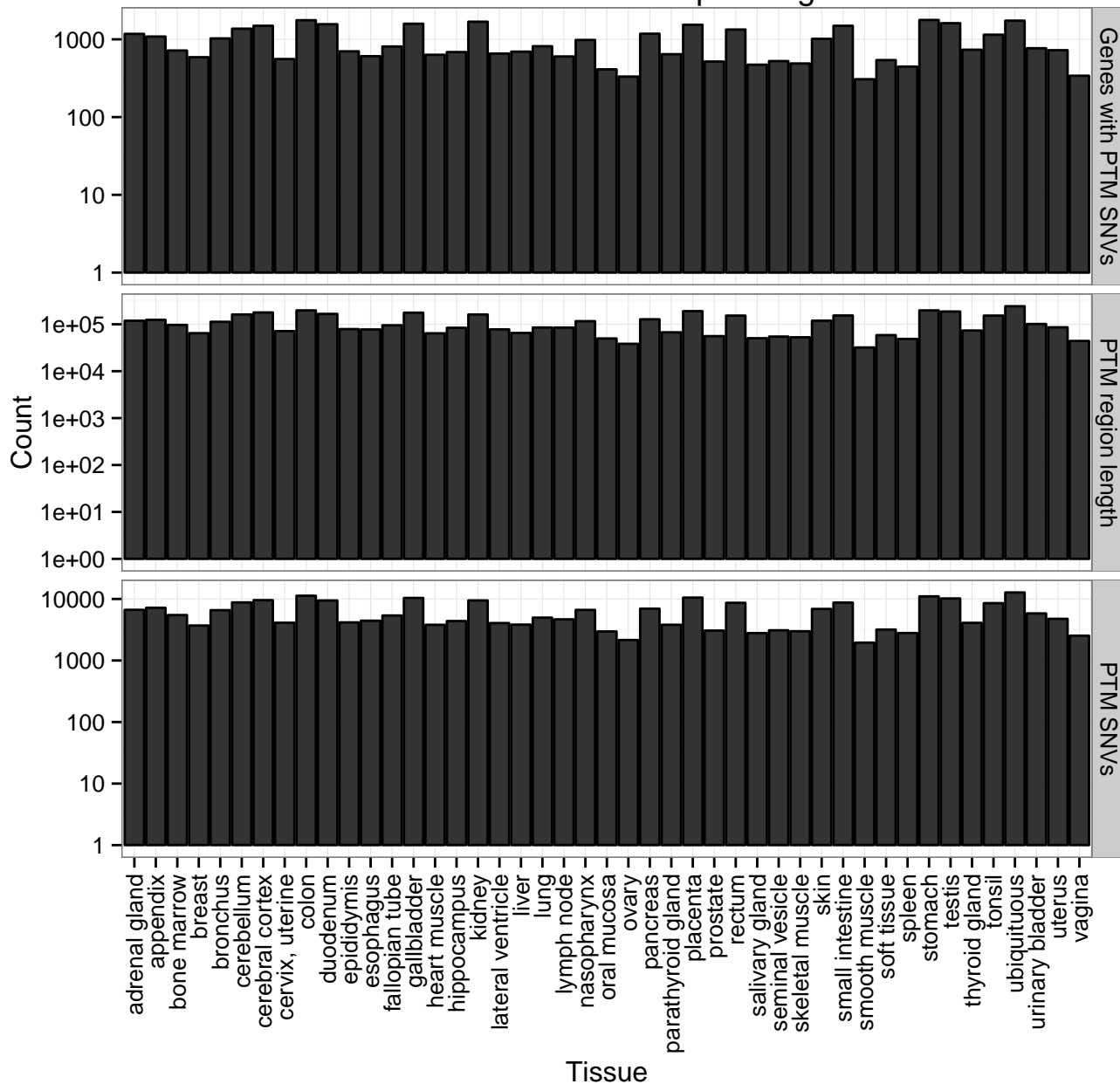

Supplement: S19 Fig — (PDF) [file pgen.1004919.s021.pdf]

Number of processes and pathways with specific selection in PTM sites (FDR  $p < 0.05$ )

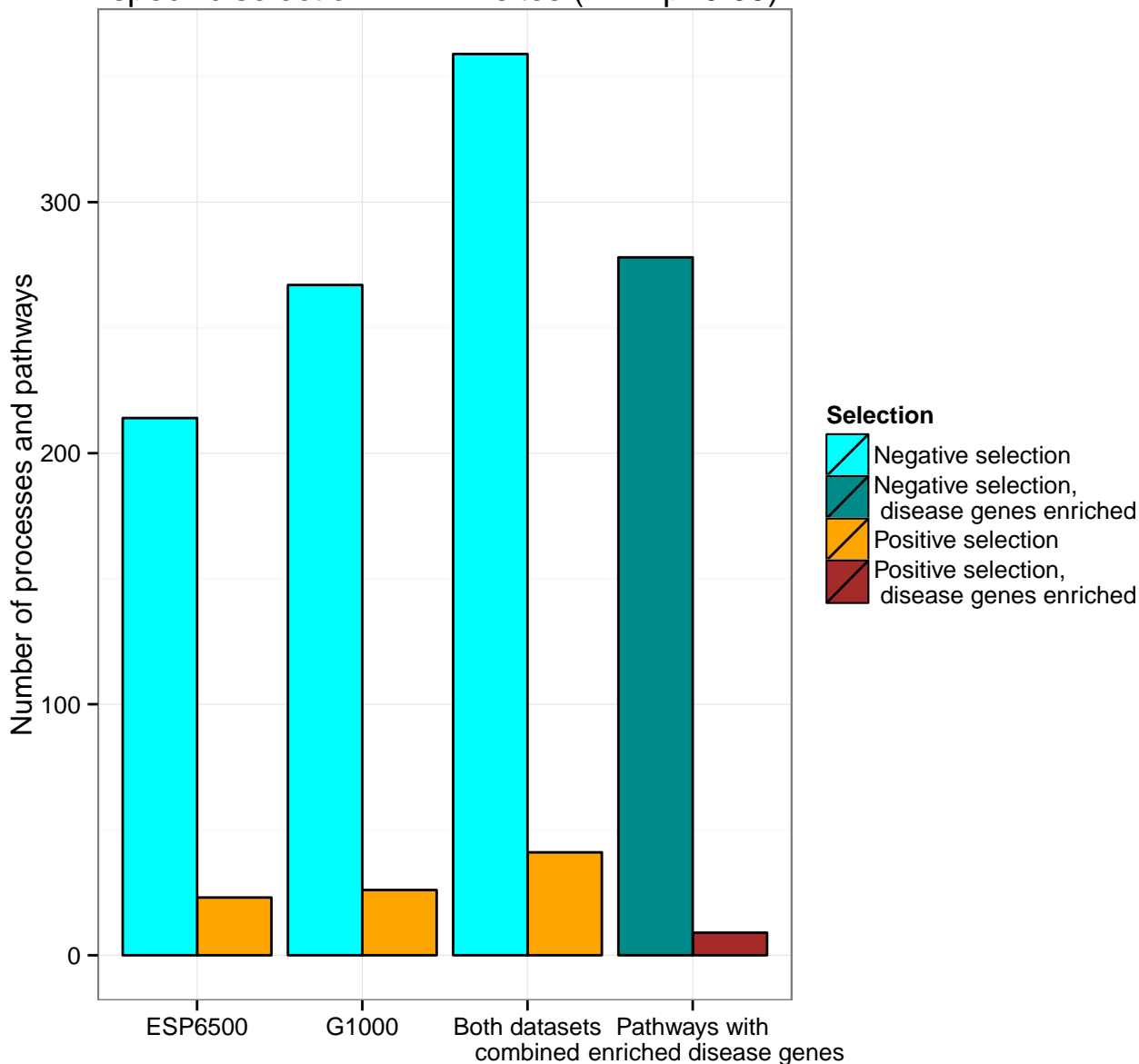

Supplement: S20 Fig — Selection was measured with logistic regression tests with protein disorder as confounding factor. We tested 9,084 biological processes (GO), pathways (KEGGm Reactome), and protein complexes (CORUM). Enrichment of disease genes with PTM mutations was computed with Fisher’s exact test (FDR p<0.01). (PDF) [file pgen.1004919.s022.pdf]

# PTM statistics in pathway analysis

Genes with PTM SNVs

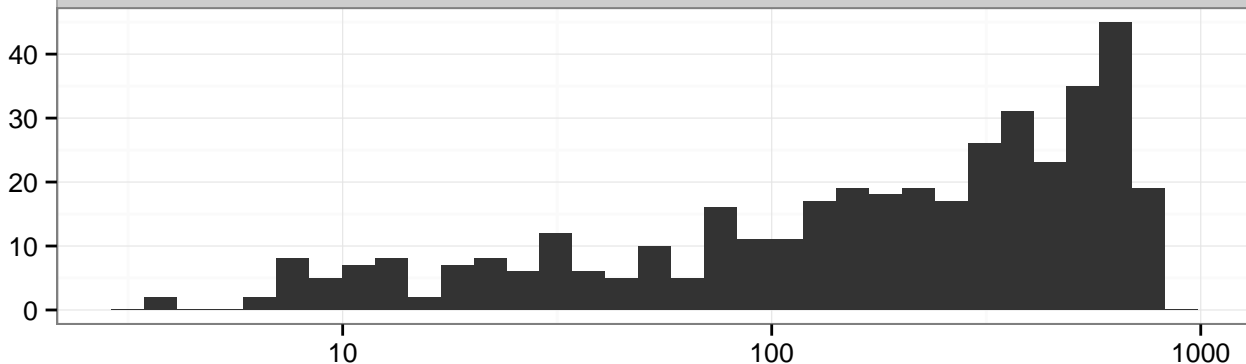

PTM region size

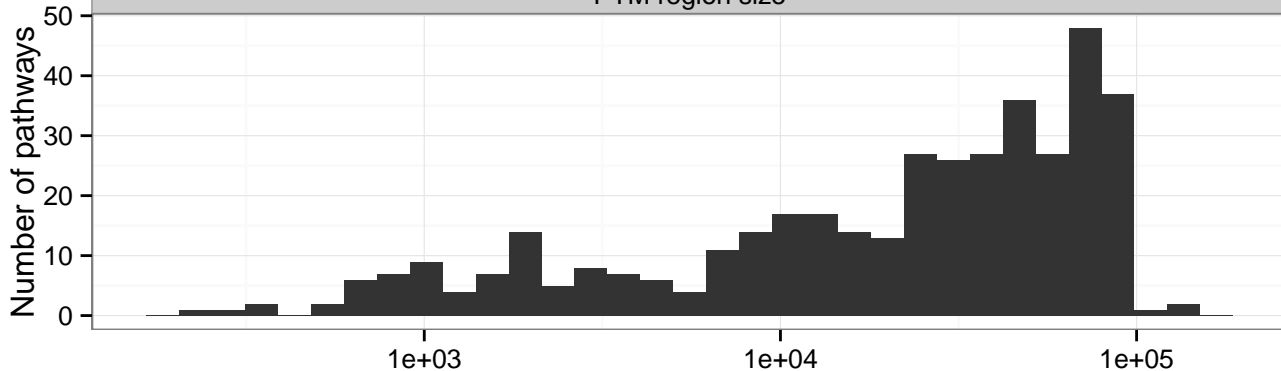

PTM SNVs

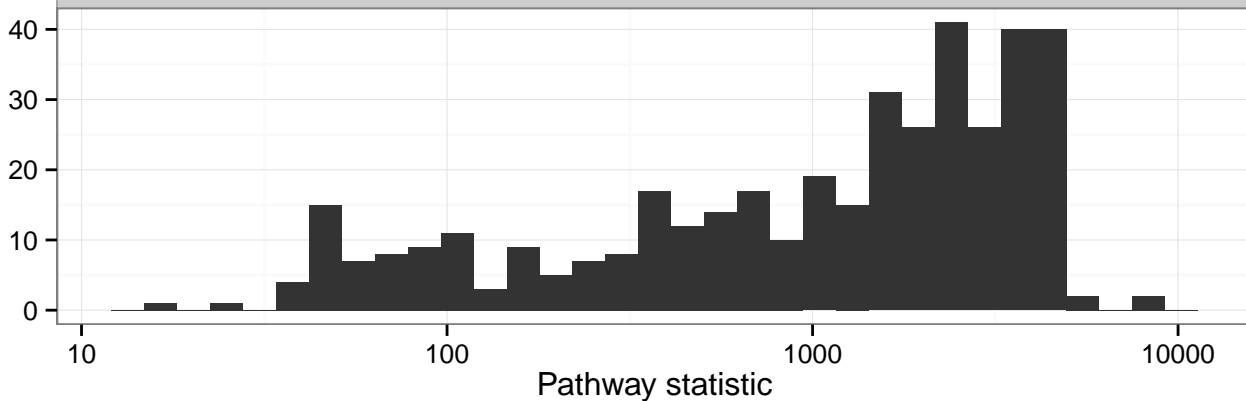

Supplement: S21 Fig — (PDF) [file pgen.1004919.s023.pdf]

# PTM statistics by modification types and proximity

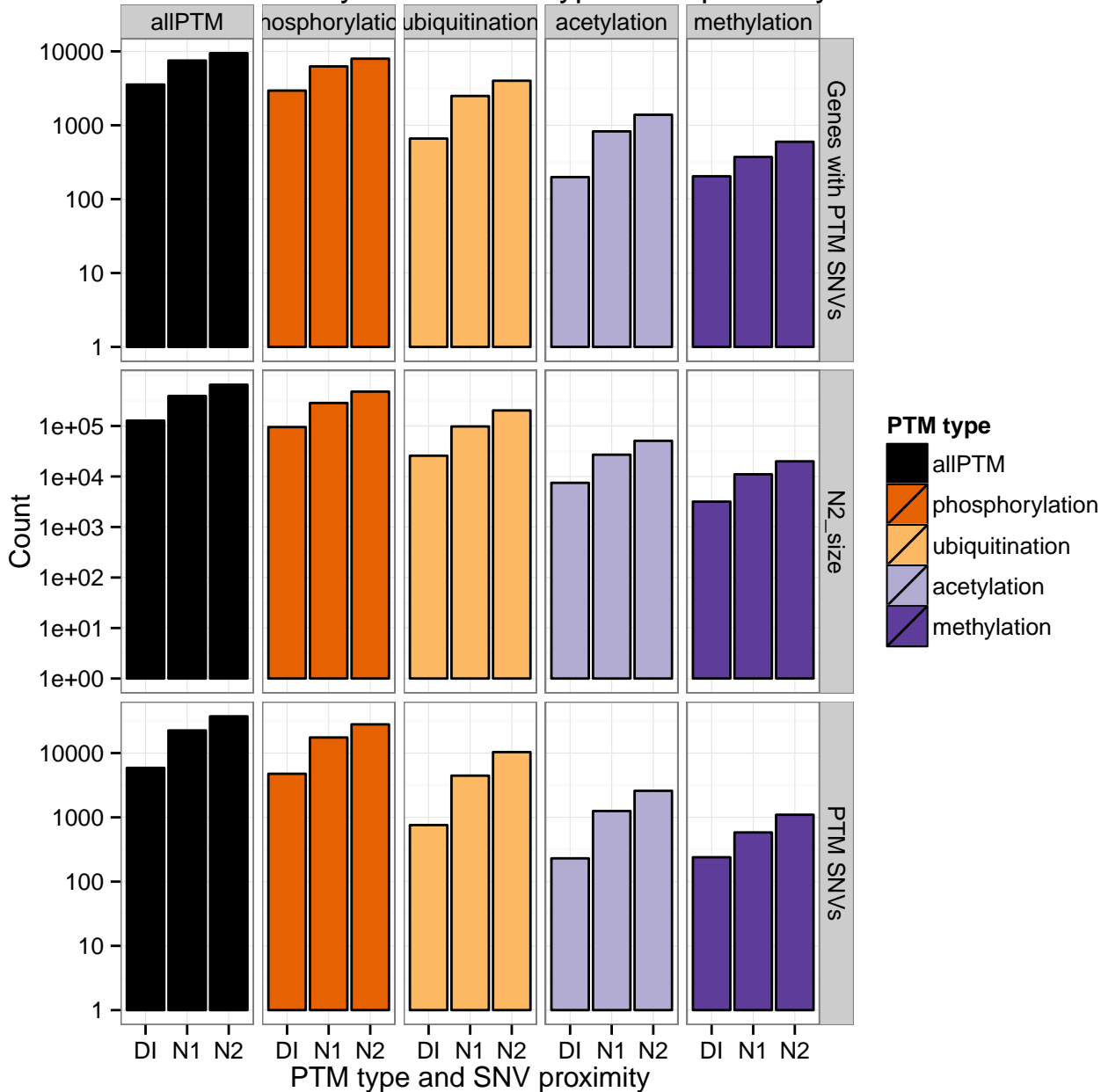

Supplement: S22 Fig — (PDF) [file pgen.1004919.s024.pdf]

PTM sites per region (total n=55543)

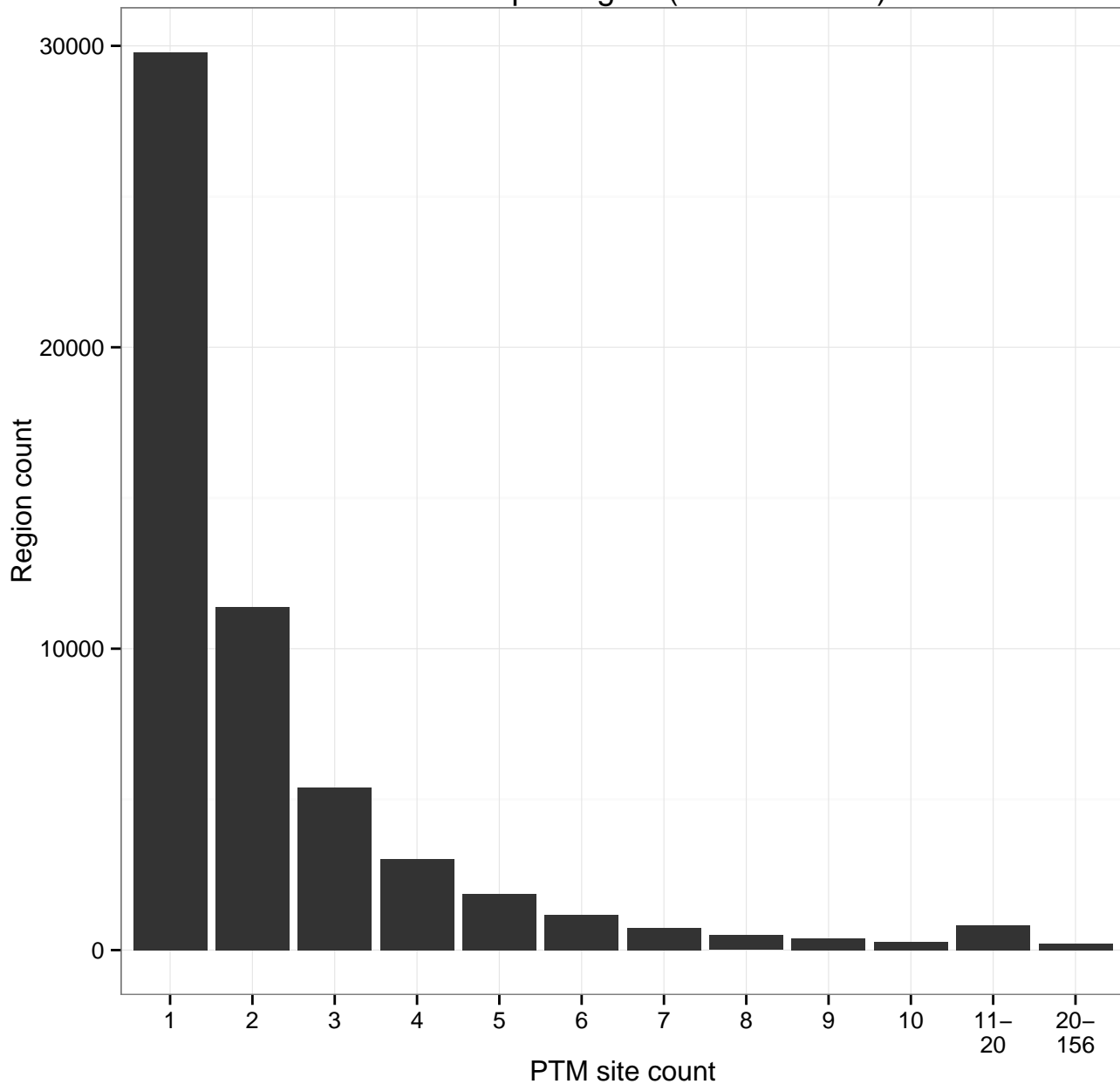

Supplement: S23 Fig — 46% of PTM regions have more than one site. (PDF) [file pgen.1004919.s025.pdf]

# PTM statistics in clustered modification sites

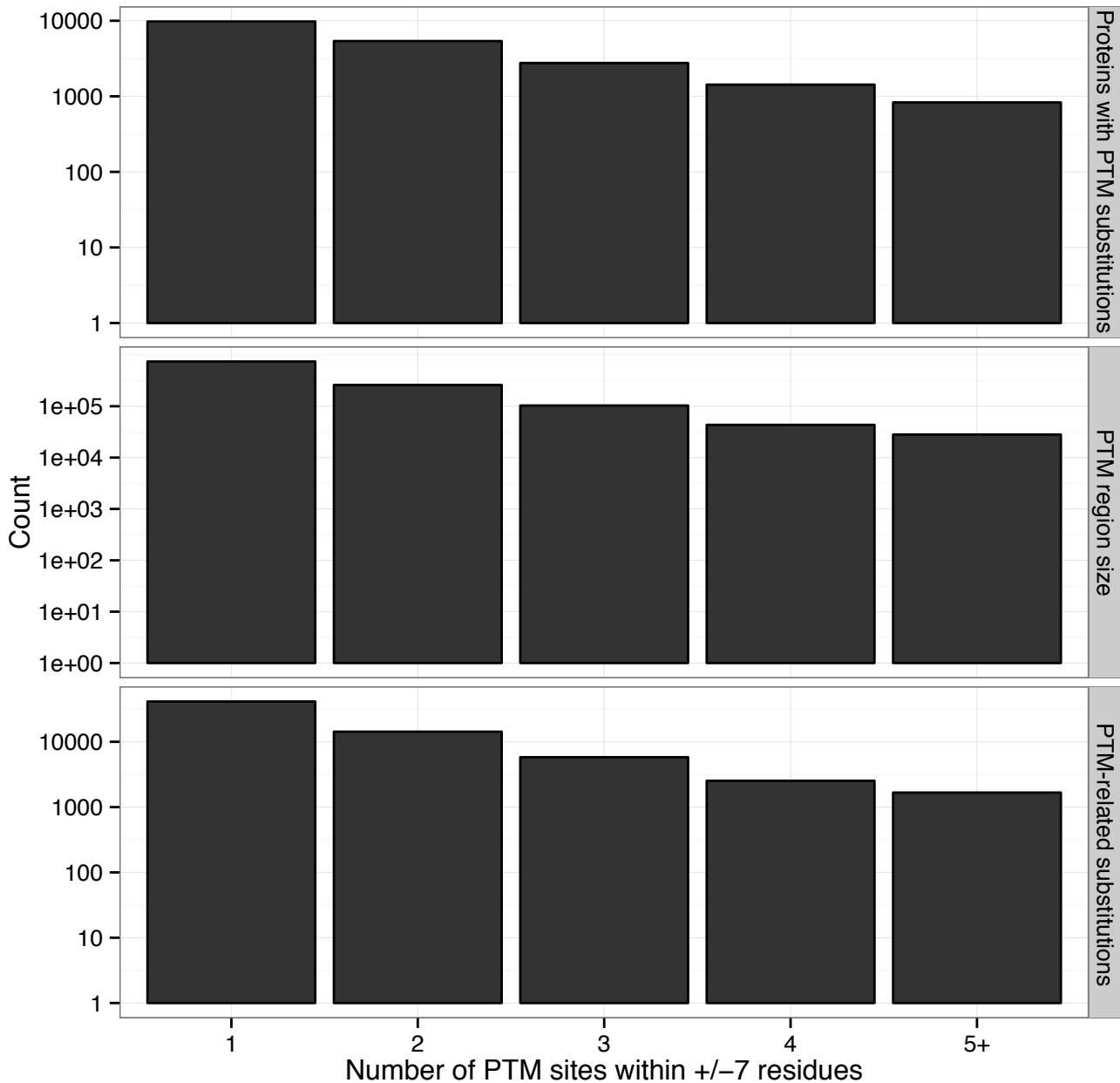

Supplement: S24 Fig — (PDF) [file pgen.1004919.s026.pdf]

PTM statistics of kinase motif breaker (MB) sites

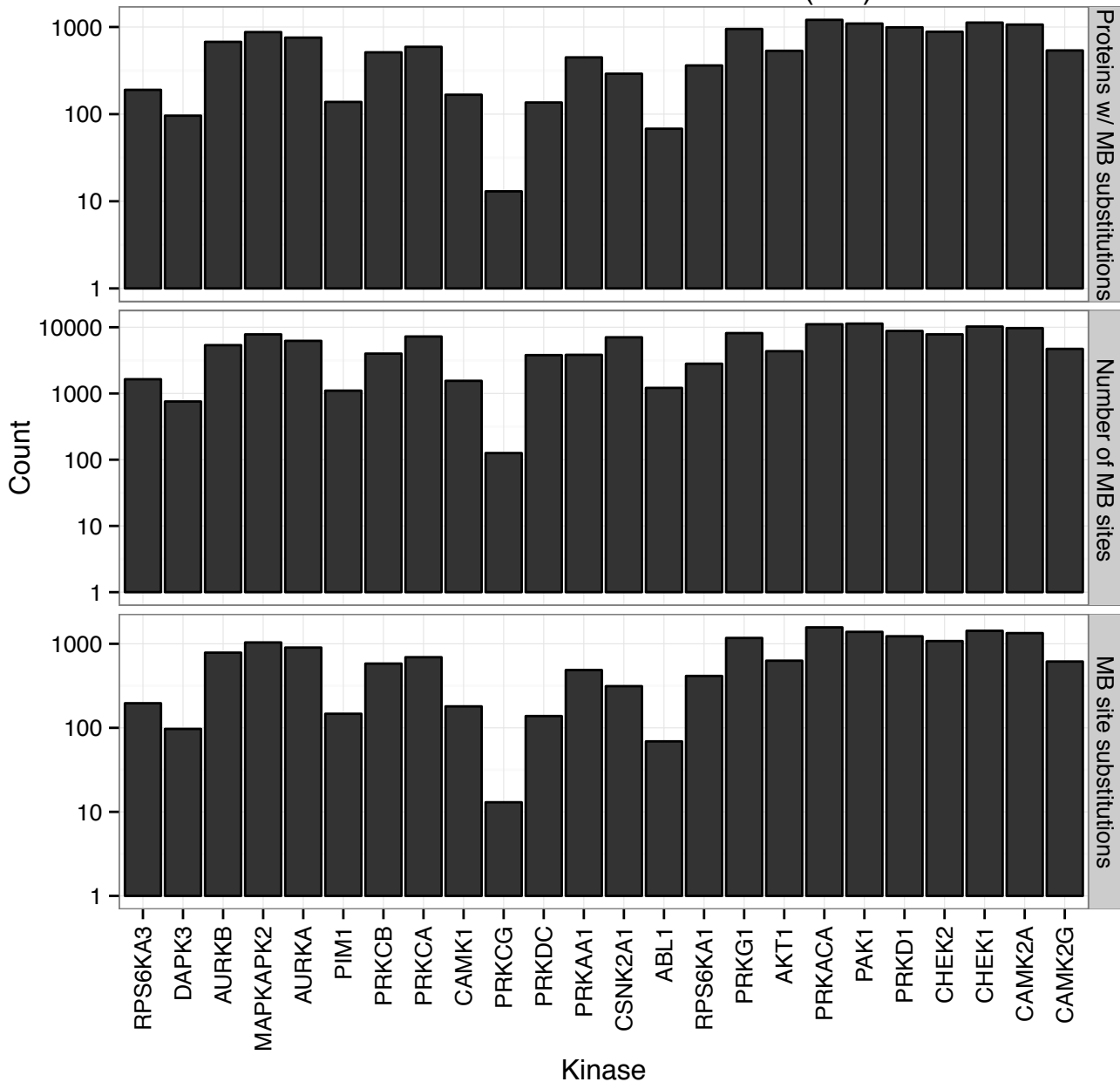

Supplement: S26 Fig — (PDF) [file pgen.1004919.s028.pdf]

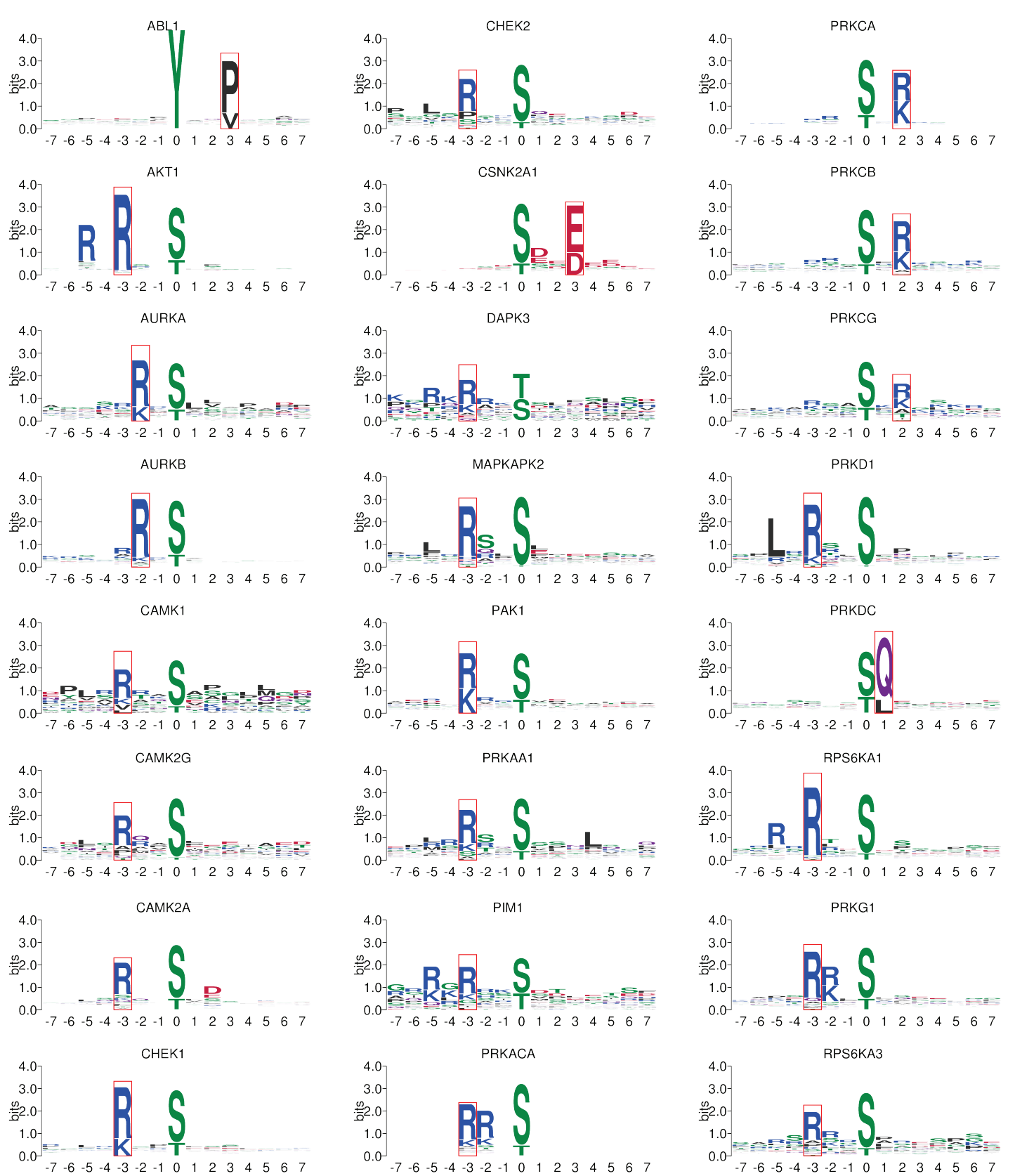

Supplement: S27 Fig — Protein residues in motif-breaker sites are highlighted with red boxes. (PDF) [file pgen.1004919.s029.pdf]
