# Supplementary material for: Evolutionary Constraint and Disease Associations of Post-Translational Modification Sites in Human Genomes
Source: PLoS Genet. 2015 Jan 22;11(1):e1004919. doi: 10.1371/journal.pgen.1004919 (PMC4303425; doi:10.1371/journal.pgen.1004919)
Supplement: S4 Fig — Top: Fraction of rare substitutions in PTM regions compared to non-PTM protein sequences. Bottom: ratio of non-synonymous to synonymous variants in PTM regions vs non-PTM protein sequence. P-values are computed using paired Wilcoxon tests across bins representing 1% of proteins with matched variation. (PDF) [file pgen.1004919.s006.pdf]

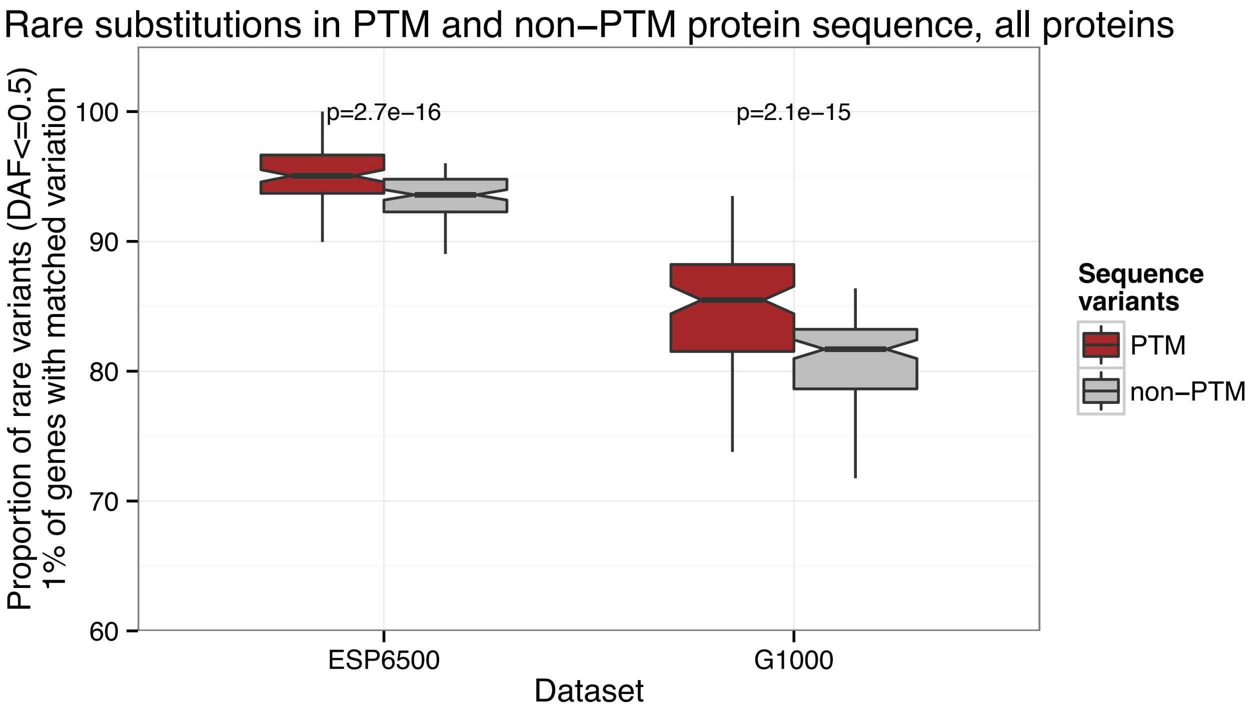

Rare substitutions in PTM and non−PTM protein sequence, all proteins
Proportion of rare variants (DAF<=0.5)
1% of genes with matched variation
p=2.7e−16
p=2.1e−15
100
90
80
70
60
ESP6500
G1000
Dataset
Sequence variants
PTM
non−PTM

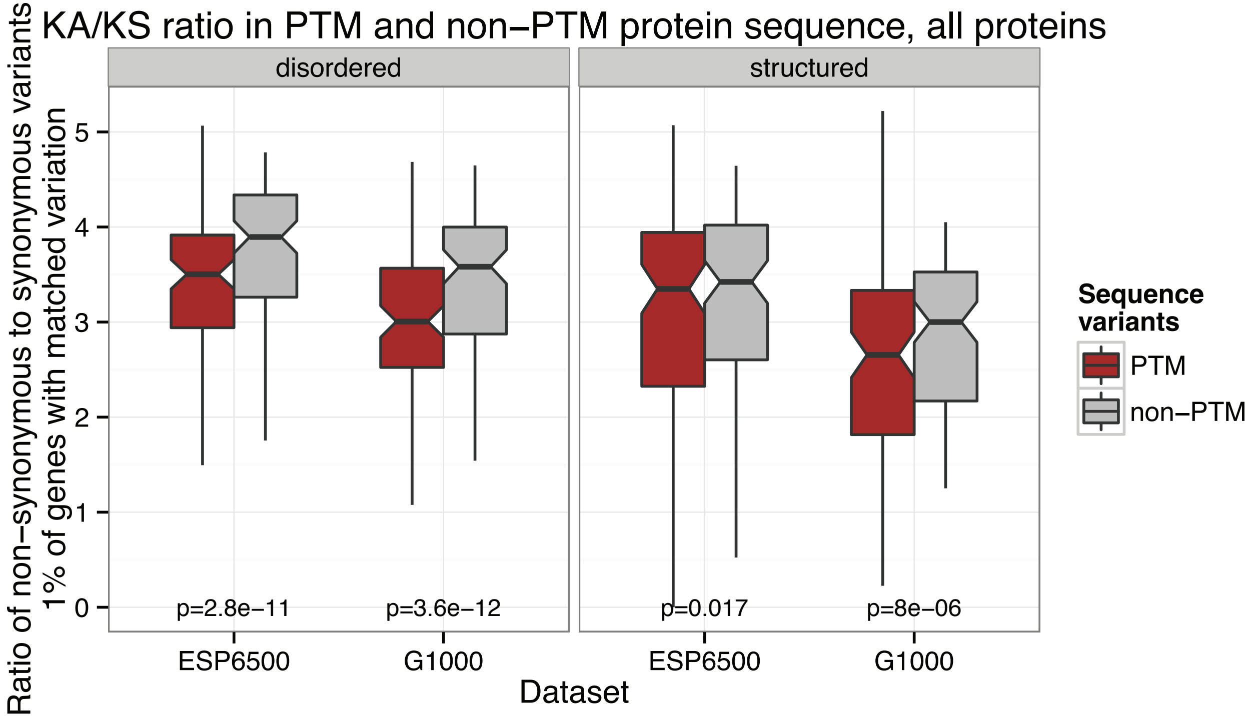

KA/KS ratio in PTM and non−PTM protein sequence, all proteins
Ratio of non−synonymous to synonymous variants
1% of genes with matched variation
disordered
structured
5
4
3
2
1
0
p=2.8e−11
p=3.6e−12
p=0.017
p=8e−06
ESP6500
G1000
ESP6500
G1000
Dataset
Sequence variants
PTM
non−PTM
