# Supplementary material for: Evolutionary Constraint and Disease Associations of Post-Translational Modification Sites in Human Genomes
Source: PLoS Genet. 2015 Jan 22;11(1):e1004919. doi: 10.1371/journal.pgen.1004919 (PMC4303425; doi:10.1371/journal.pgen.1004919)
Supplement: S5 Fig — PTM sites were filtered based on number of associated publications (xPMID—X or more PubMed IDs). Left: Fraction of rare substitutions in PTM regions compared to non-PTM protein sequences. Right: ratio of non-synonymous to synonymous variants in PTM regions compared to non-PTM protein sequence. (PDF) [file pgen.1004919.s007.pdf]

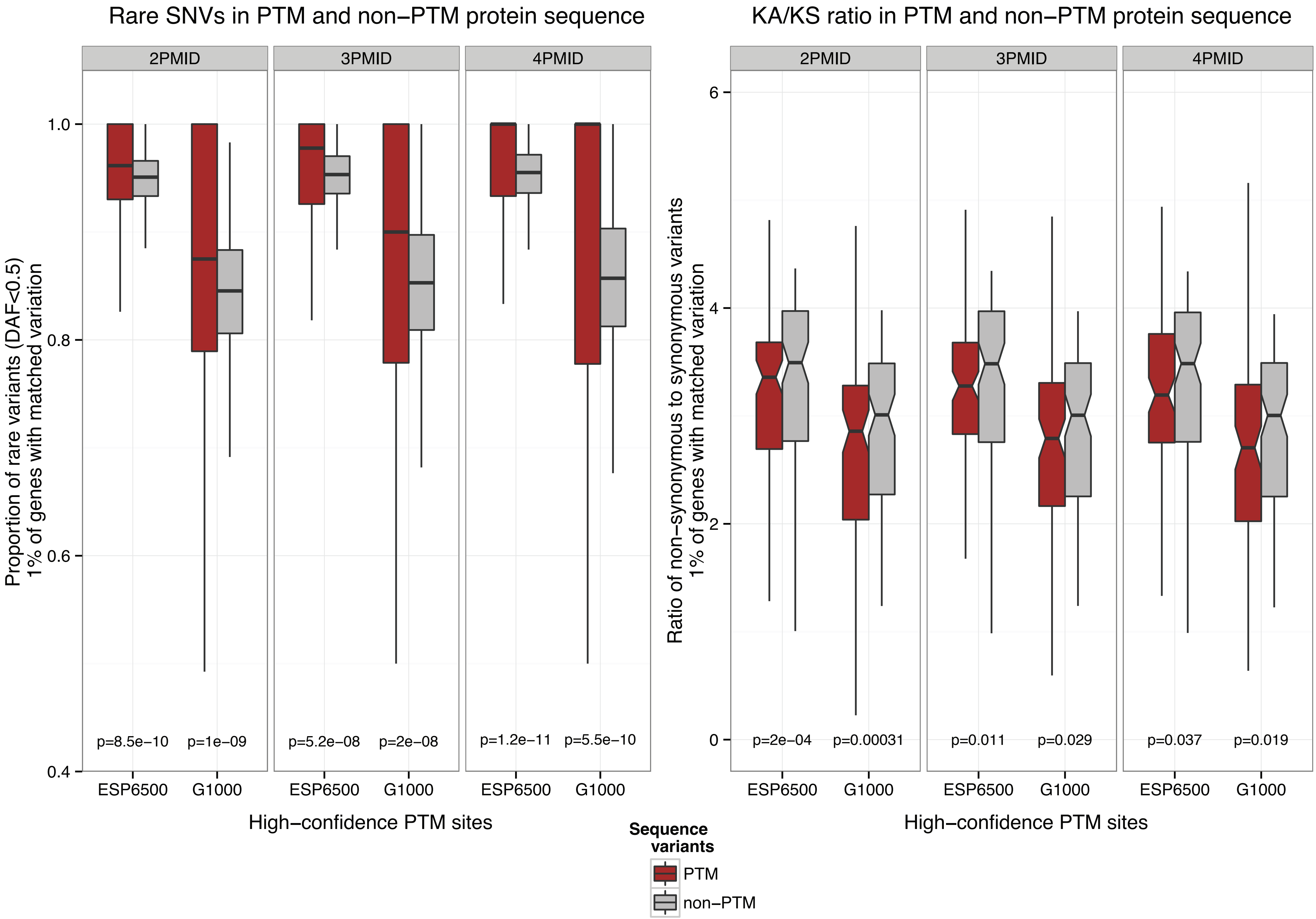
Rare SNVs in PTM and non−PTM protein sequence
KA/KS ratio in PTM and non−PTM protein sequence
2PMID
3PMID
4PMID
Proportion of rare variants (DAF<0.5)
1% of genes with matched variation
1.0
0.8
0.6
0.4
p=8.5e−10
p=1e−09
p=5.2e−08
p=2e−08
p=1.2e−11
p=5.5e−10
ESP6500
G1000
High−confidence PTM sites
Ratio of non−synonymous to synonymous variants
1% of genes with matched variation
6
4
2
0
p=2e−04
p=0.00031
p=0.011
p=0.029
p=0.037
p=0.019
Sequence variants
PTM
non−PTM
