# Supplementary material for: Evolutionary Constraint and Disease Associations of Post-Translational Modification Sites in Human Genomes
Source: PLoS Genet. 2015 Jan 22;11(1):e1004919. doi: 10.1371/journal.pgen.1004919 (PMC4303425; doi:10.1371/journal.pgen.1004919)
Supplement: S7 Fig — Variation is quantified by number of substituted protein residues per total protein sequence. Each bar represents 1% of proteins with similar variation. Y-axis shows log2 ratios of Ka/Ks ratios in PTM regions over non-PTM protein sequence. Panels represent Ka/Ks ratios in all sequence (top), and separately for structured (middle) and disordered sequence (bottom). (PDF) [file pgen.1004919.s009.pdf]

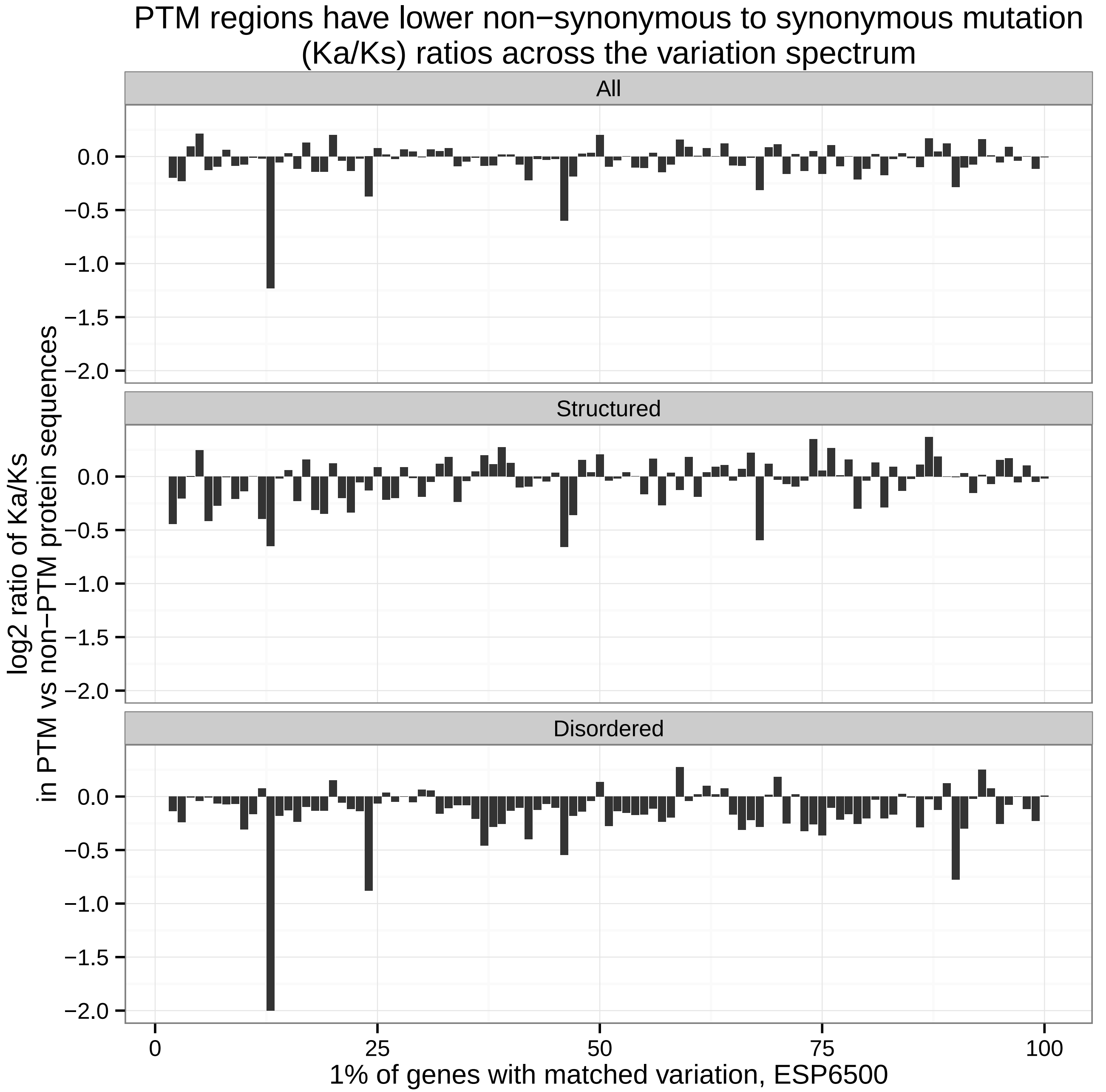

PTM regions have lower non−synonymous to synonymous mutation (Ka/Ks) ratios across the variation spectrum
All
Structured
Disordered
log2 ratio of Ka/Ks in PTM vs non−PTM protein sequences
0.0
−0.5
−1.0
−1.5
−2.0
0
25
50
75
100
1% of genes with matched variation, ESP6500
