# Supplementary material for: Evolutionary Constraint and Disease Associations of Post-Translational Modification Sites in Human Genomes
Source: PLoS Genet. 2015 Jan 22;11(1):e1004919. doi: 10.1371/journal.pgen.1004919 (PMC4303425; doi:10.1371/journal.pgen.1004919)
Supplement: S10 Fig — Top: fraction of rare substitutions in PTM regions and variation-matched non-PTM regions. Bottom: Ka/Ks ratio in PTM regions relative to matched protein-coding sequence. P-values are computed with paired Wilcoxon tests. (PDF) [file pgen.1004919.s012.pdf]

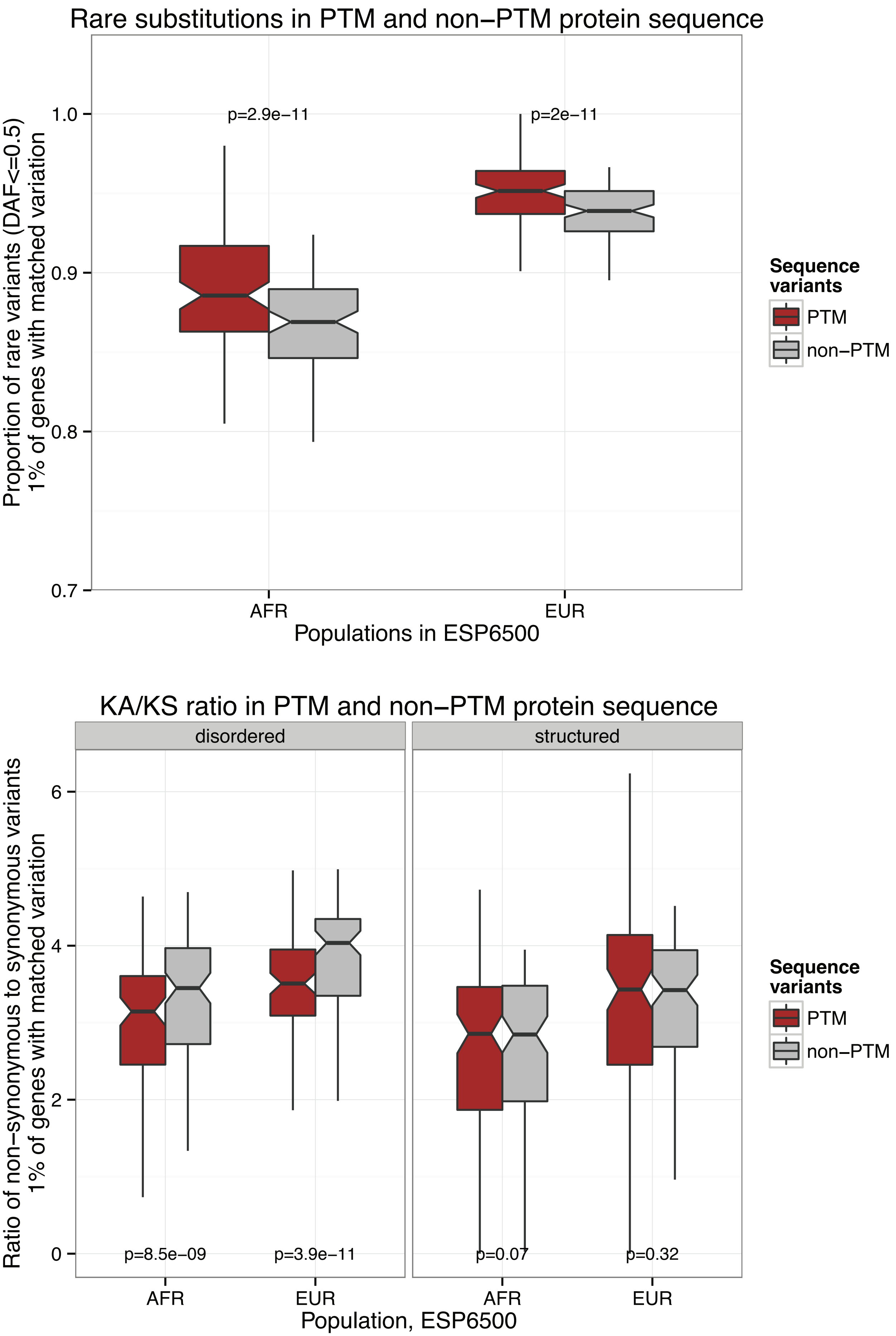
Rare substitutions in PTM and non−PTM protein sequence
Proportion of rare variants (DAF<=0.5)
1% of genes with matched variation
1.0
0.9
0.8
0.7
p=2.9e−11
p=2e−11
AFR
EUR
Populations in ESP6500
Sequence variants
PTM
non−PTM
KA/KS ratio in PTM and non−PTM protein sequence
disordered
structured
Ratio of non−synonymous to synonymous variants
1% of genes with matched variation
6
4
2
0
p=8.5e−09
p=3.9e−11
p=0.07
p=0.32
AFR
EUR
AFR
EUR
Population, ESP6500
Sequence variants
PTM
non−PTM
