# Supplementary material for: Evolutionary Constraint and Disease Associations of Post-Translational Modification Sites in Human Genomes
Source: PLoS Genet. 2015 Jan 22;11(1):e1004919. doi: 10.1371/journal.pgen.1004919 (PMC4303425; doi:10.1371/journal.pgen.1004919)
Supplement: S25 Fig — Letter size and color indicates frequency of mutation annotation in the HGMD database. Clouds are not drawn to scale. (PDF) [file pgen.1004919.s027.pdf]

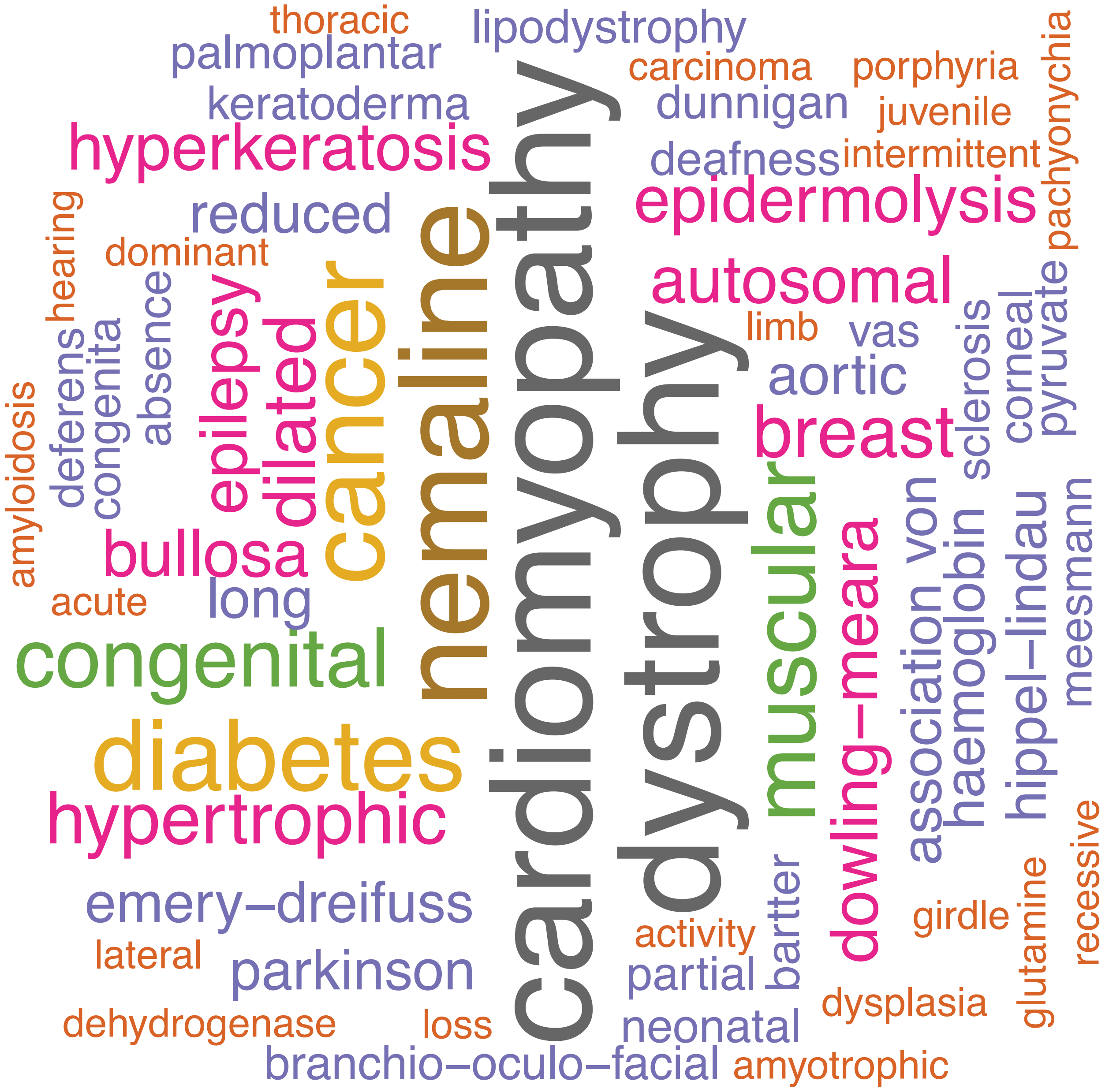

thoracic
lipodystrophy
palmoplantar
carcinoma
porphyria
pachyonychia
keratoderma
dunnigan
juvenile
hyperkeratosis
deafness
intermittent
epidermolysis
reduced
hearing
dominant
autosomal
limb
vas
aortic
breast
sclerosis
corneal
pyruvate
defferens
congenita
absence
epilepsy
dilated
cancer
nemaline
cardiomyopathy
dystrophy
muscular
amyloidosis
bullosa
acute
long
congenital
dowling–meara
association von
haemoglobin
hippel–lindau
meesmann
diabetes
hypertrophic
emery–dreifuss
lateral
parkinson
activity
partial
bartter
girdle
glutamine
recessive
dehydrogenase
loss
neonatal
dysplasia
branchio–oculo–facial
amyotrophic

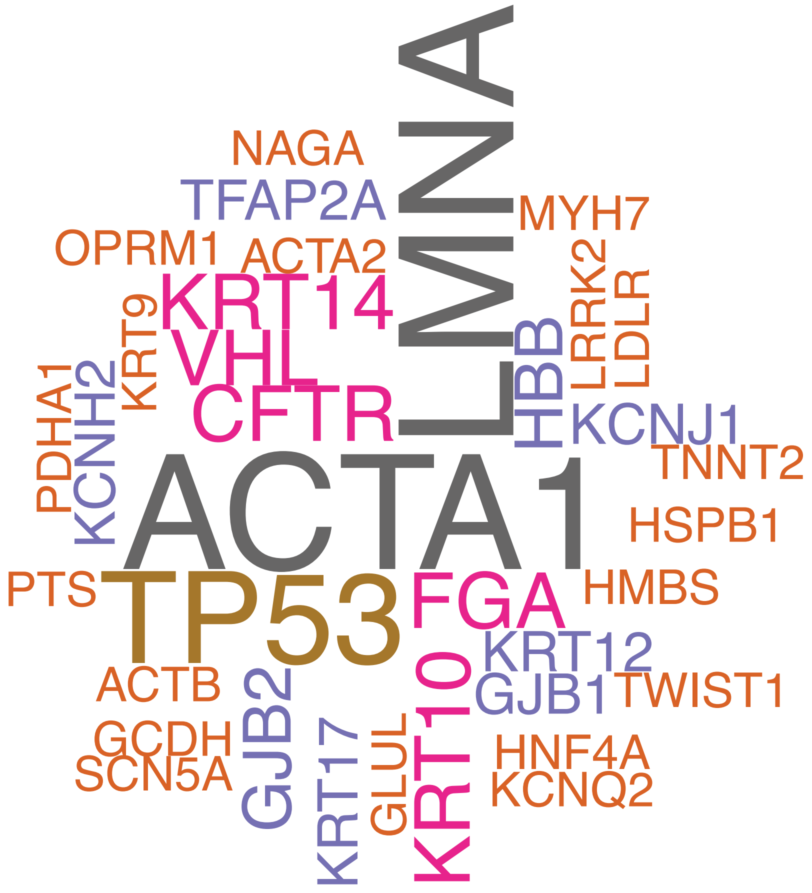

LMNA
NAGA
TFAP2A
MYH7
OPRM1
ACTA2
KRT14
KRT9
LRRK2
LDLR
VHL
PDHA1
KCNH2
CFTR
HBB
KCNJ1
TNNT2
ACTA1
HSPB1
PTS
TP53
FGA
HMBS
KRT12
ACTB
GJB2
KRT10
GJB1
TWIST1
GCDH
SCN5A
KRT17
GLUL
HNF4A
KCNQ2
