# Supplementary material for: Evolutionary Constraint and Disease Associations of Post-Translational Modification Sites in Human Genomes
Source: PLoS Genet. 2015 Jan 22;11(1):e1004919. doi: 10.1371/journal.pgen.1004919 (PMC4303425; doi:10.1371/journal.pgen.1004919)
Supplement: S28 Fig — Letter size and color indicates number of disease annotations. (PDF) [file pgen.1004919.s030.pdf]

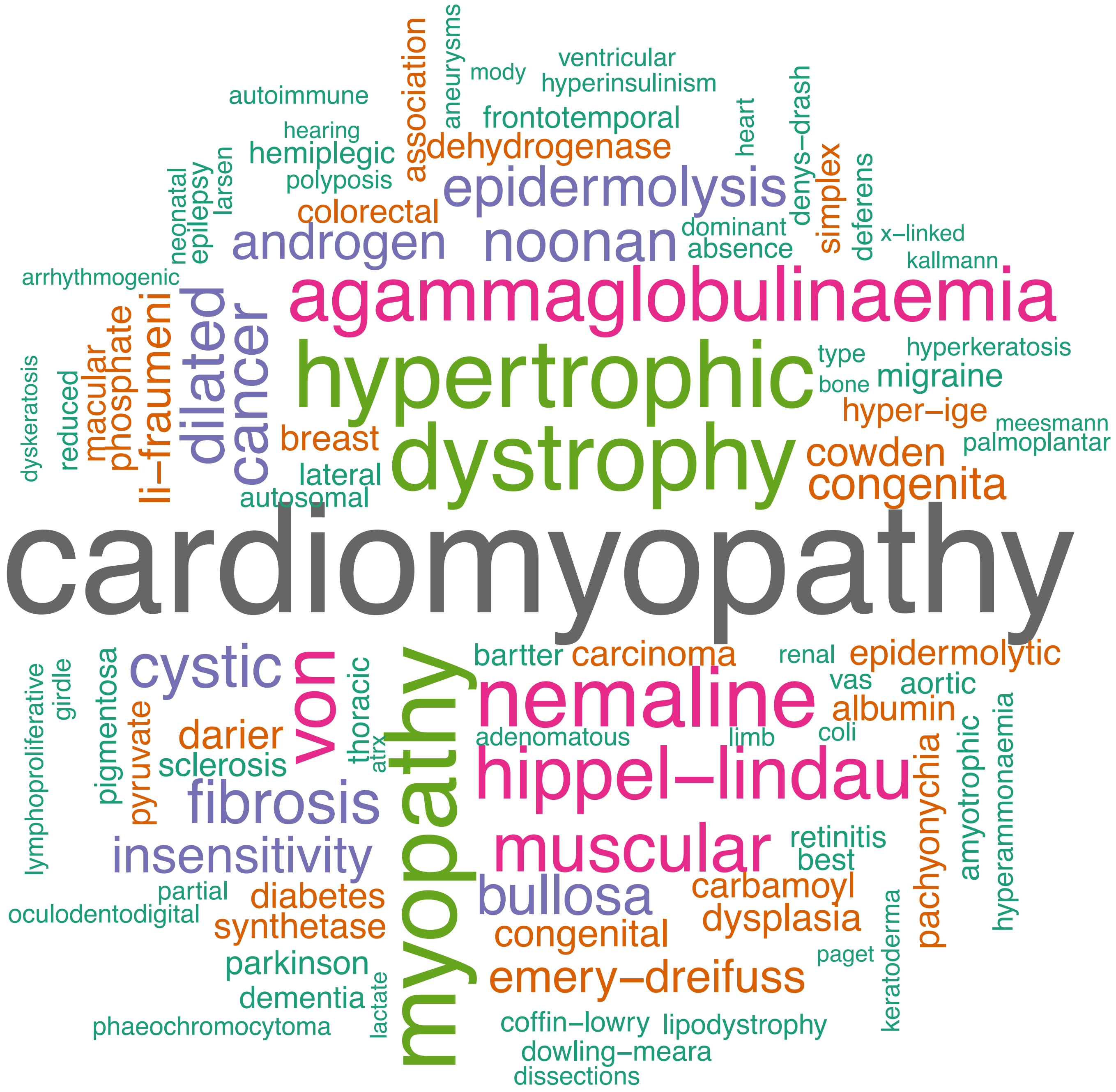
aneurysms
association
ventricular
mody
hyperinsulinism
autoimmune
hearing
frontotemporal
heart
denys–drash
dehydrogenase
hemiplegic
simplex
polyposis
larsen
neonatal
epilepsy
epidermolysis
deferens
colorectal
androgen
noonan
dominant
absence
x–linked
kallmann
arrhythmogenic
agammaglobulinaemia
dyskeratosis
reduced
macular
phosphate
li–fraumeni
dilated
cancer
hypertrophic
type
bone
hyperkeratosis
migraine
hyper–ige
meesmann
palmoplantar
breast
dystrophy
cowden
lateral
autosomal
congenita
cardiomyopathy
lymphoproliferative
girdle
pigmentosa
cystic
von
thoracic
myopathy
bartter
carcinoma
renal
epidermolytic
vas
aortic
nemaline
albumin
pyruvate
darier
atrx
adenomatous
limb
coli
sclerosis
hippel–lindau
pachyonychia
amyotrophic
hyperammonaemia
fibrosis
insensitivity
muscular
retinitis
best
partial
diabetes
bullosa
carbamoyl
oculodentodigital
synthetase
dysplasia
congenital
keratoderma
paget
parkinson
emery–dreifuss
dementia
lactate
phaeochromocytoma
coffin–lowry
lipodystrophy
dowling–meara
dissections
